# Phylogenomic analysis of proteins that are distinctive of *Archaea *and its main subgroups and the origin of methanogenesis

**DOI:** 10.1186/1471-2164-8-86

**Published:** 2007-03-29

**Authors:** Beile Gao, Radhey S Gupta

**Affiliations:** 1 Department of Biochemistry and Biomedical Science, McMaster University, Hamilton, L8N3Z5, Canada

## Abstract

**Background:**

The Archaea are highly diverse in terms of their physiology, metabolism and ecology. Presently, very few molecular characteristics are known that are uniquely shared by either all archaea or the different main groups within archaea. The evolutionary relationships among different groups within the Euryarchaeota branch are also not clearly understood.

**Results:**

We have carried out comprehensive analyses on each open reading frame (ORFs) in the genomes of 11 archaea (3 Crenarchaeota – *Aeropyrum pernix, Pyrobaculum aerophilum *and *Sulfolobus acidocaldarius*; 8 Euryarchaeota – *Pyrococcus abyssi, Methanococcus maripaludis, Methanopyrus kandleri, Methanococcoides burtonii, Halobacterium *sp. NCR-1, *Haloquadratum walsbyi*, *Thermoplasma acidophilum *and *Picrophilus torridus*) to search for proteins that are unique to either all Archaea or for its main subgroups. These studies have identified 1448 proteins or ORFs that are distinctive characteristics of Archaea and its various subgroups and whose homologues are not found in other organisms. Six of these proteins are unique to all Archaea, 10 others are only missing in *Nanoarchaeum equitans *and a large number of other proteins are specific for various main groups within the Archaea (e.g. Crenarchaeota, Euryarchaeota, Sulfolobales and Desulfurococcales, Halobacteriales, Thermococci, Thermoplasmata, all methanogenic archaea or particular groups of methanogens). Of particular importance is the observation that 31 proteins are uniquely present in virtually all methanogens (including *M. kandleri*) and 10 additional proteins are only found in different methanogens as well as *A. fulgidus*. In contrast, no protein was exclusively shared by various methanogen and any of the Halobacteriales or Thermoplasmatales. These results strongly indicate that all methanogenic archaea form a monophyletic group exclusive of other archaea and that this lineage likely evolved from *Archaeoglobus*. In addition, 15 proteins that are uniquely shared by *M. kandleri *and Methanobacteriales suggest a close evolutionary relationship between them. In contrast to the phylogenomics studies, a monophyletic grouping of archaea is not supported by phylogenetic analyses based on protein sequences.

**Conclusion:**

The identified archaea-specific proteins provide novel molecular markers or signature proteins that are distinctive characteristics of *Archaea *and all of its major subgroups. The species distributions of these proteins provide novel insights into the evolutionary relationships among different groups within Archaea, particularly regarding the origin of methanogenesis. Most of these proteins are of unknown function and further studies should lead to discovery of novel biochemical and physiological characteristics that are unique to either all archaea or its different subgroups.

## Background

Archaea are widely regarded as one of the three main domains of life [1-7], although their origin is a subject of debate [8-14]. Archaeal species were earlier believed to inhabit only extreme environments such as extremely hot, or hot and acidic, extremely saline, or very acidic or alkaline conditions [15-19]. However, recent studies provide evidence that they are widespread in different environments [3,20]. The archaea also include methanogens, which grow under strictly anaerobic and often thermophilic conditions, and are the only organisms that derive all of their metabolic energy by reduction of CO_2 _by hydrogen to produce methane [21,22]. The archaeal species branch distinctly from all other organisms in phylogenetic trees based on 16S rRNA and many other gene/protein sequences [2,7,23-25]. In addition, many morphological or physiological characteristics such as the presence of branched-chain ether-linked lipids in their cell membrane, lack of peptidoglycan in their cell wall, characteristic subunit pattern of RNA polymerase, presence of modified bases in tRNA, presence of a unique form of DNA polymerase, have been previously indicated as defining characteristics of archaea [1,15]. However, as noted by Walsh and Doolittle [26], many of these features are either not shared by all archaea or they are also present in various eukaryotes or some thermophilic bacteria, indicating that they do not constitute distinctive characteristics of all Archaea.

The phylogenetic analyses of Archaea have led to their division into two major groups or phyla designated as Crenarchaeota and Euryarchaeota [1,2,7,13,27-29]. The Crenarchaeota species have also been referred to as 'Eocytes' by Lake and coworkers [30,31]. The species from both these groups, particularly Euryarchaeota, are highly diverse in terms of their metabolism and physiology. Based on their metabolic and physiological characteristics and other unique features, five functionally distinct groups within Euryarchaeota are currently recognized: methanogens, sulfate reducers, extreme halophiles, cell wall-less archaea, and extremely thermophilic sulfur metabolizing archaea [2,13,32]. Some of these groups, such as methanogens, are polyphyletic in different phylogenetic trees [13,33,34]. However, the sets of genes or proteins that are unique to these different functional groups and distinguish them from all others remain to be identified. In the past 10 years, complete genomes of many archaeal species (29 at the time when these analyses were completed) covering all major divisions within the Archaea have been sequenced (see Table 1). Comparative analyses of these sequences provide a valuable resource for identifying different genes/proteins that are distinctive characteristics of various taxonomic and functional groups within Archaea [27,35-37].

Whole proteins that are uniquely present in particular groups or subgroups of organisms but not found anywhere else provide valuable molecular markers for taxonomic, phylogenetic and biochemical studies. These proteins, which we refer to as signature proteins in our work, and others have called them as ORFans or conserved hypothetical proteins, are present at different phylogenetic depths, such as genus, family, order or even phylum [35,36,38-42]. In our recent work, a large number of such proteins that are distinctive characteristics of several groups within bacteria (viz. α-proteobacteria, ε-proteobacteria, Chlamydia and Actinobacteria), and also their subgroups, were identified [39-43]. These proteins provide not only valuable molecular markers for identifying and circumscribing species belonging to these major groups (and their subgroups) in molecular terms, but their species distribution pattern also provides useful information about the branching order within these groups. As archaea constitute a very diverse group, identification of sets of proteins that are specific for its main groups and subgroups should prove useful in terms of identifying molecular characteristics that are unique to them. Additionally, this information should also be helpful in understanding the evolutionary relationships among different groups.

Comparative studies on limited numbers of archaeal genomes have been carried out by a number of investigators using different criteria. Graham et al. [36] analyzed 9 archaeal genomes to identify signature proteins that function uniquely within the Archaea. Their definition of an archaeal signature protein required it to be present in only two different euryarchaeal species and they identified 353 archaeal signature proteins. Makarova and Koonin [27,35] have analyzed archaeal genomes to identify core sets of genes, which are present in all archaeal species, but which are not restricted to the archaeal species. Recently, Walsh and Doolittle have analyzed prokaryotic genomes to measure dissimilarity between Archaea and Bacteria [26]. Although it was reported that 28% of the proteins from archaeal genomes are restricted to the Archaea, specific proteins that were present in different groups of archaea were not identified. Other comparative studies using different criteria have been conducted on smaller groups within archaea such as *Pyrococcus*, *Sulfolobus *and thermoacidophilic organisms (to be discussed later). However, thus far no comprehensive phylogenomics study on different archaeal genomes has been carried out using the same standard criteria to identify proteins or ORFs that are shared by all archaea or its different major lineages. In this study we have carried out comparative analyses of archaeal genomes using uniform criteria to identify proteins that are uniquely present in archaeal species at different phylogenetic depths (genus or higher) representing all major groups within the Archaea.

## Results and discussion

### A. Phylogenetic analyses of archaeal species

Prior to undertaking comparative studies on archaeal genomes, phylogenetic analysis of sequenced archaeal species was carried out so that the results of phylogenomics analyses could be compared with those obtained by traditional phylogenetic approaches. Phylogenetic trees for the archaeal species based on 16S rRNA as well as concatenated sequences of translation and transcription-related proteins have been published by other investigators [7,28,32,44]. In the present work, we have constructed phylogenetic trees for 29 archaeal species (see Table 1) using a set of 31 universally distributed proteins that are involved in a broad range of functions [45]. The sequence of *Haloquadratum walsbyi *DSM 16790, which became available afterward, was not included in these studies. Phylogenetic trees based on a concatenated sequence alignment of these proteins were constructed using the neighbour-joining (NJ), maximum-likelihood (ML) and maximum-parsimony (MP) methods.

**Figure 1:**
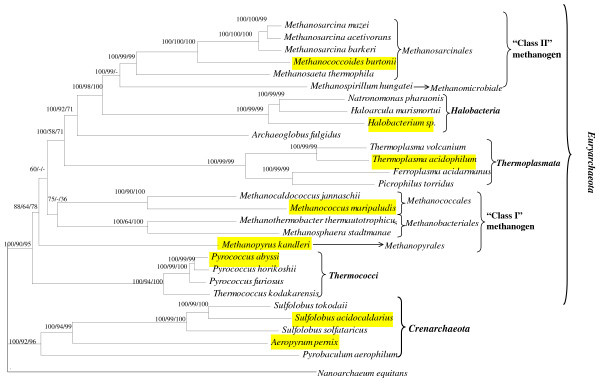
A neighbour-joining distance tree based on a concatenated sequence alignment for 31 widely distributed proteins. The numbers on the nodes indicate bootstrap scores observed in NJ/ML/MP analyses. The species shaded in yellow were selected as the query genomes for blast searches.

The results of these analyses are presented in Fig. 1. All three methods gave very similar tree topologies except for the branching positions of *M. kandleri *and *Methanospirillum hungatei*, which were found to be variable. Except for this, the branching pattern of the archaeal species based on our dataset is very similar to that reported by Gribaldo et al. [13,32] based on concatenated sequences of translation and transcription-related proteins. In the tree shown, the *Crenarchaeota *and *Euryarchaeota*, the two major phyla within *Archaea *were clearly distinguished from each other. The phylogenetic affinity of *Nanoarchaeum*, which has a long-branch length, was not resolved in this or various other trees [32,46]. Within *Crenarchaeota*, *Pyrobaculum *was indicated to be a deeper branch, and *Aeropyrum *branched in between the *Pyrobaculum *and *Sulfolobus*. Within *Euryarchaeota*, the clades corresponding to *Halobacteria, Thermococci *and *Thermoplasmata *were resolved with high bootstrap scores, but the methanogens were split into 2–3 clusters. One of these clusters that has low bootstrap score consisted of *Methanobacteriales *and *Methanococcales *with *M. kandleri *(*Methanopyrales*) branching in its vicinity [34,47,48]. The second cluster, with higher bootstrap score, showed a grouping of *Methanomicrobiales *and *Methanosarcinales*. These two clusters, which are separated by *Thermoplasmata, Archaeoglobi *and *Halobacteria*, have been referred to as Class I and Class II methanogens by Bapteste et al. [29].

### B. Phylogenomic analyses of archaeal genomes

To search for proteins (or ORFs), which are uniquely present in either all Archaea or various subgroups of them, blast searches were performed on each open reading frame (ORF) from a total of 11 archaeal genomes (see Table 1; shaded species in Fig. 1). These genomes included 3 Crenarchaeota (viz. *Aeropyrum pernix, Pyrobaculum aerophilum *and *Sulfolobus acidocaldarius*) [49-51] and 8 divergent Euryarchaeota species covering all main functional and phylogenetic groups (see Table 1 and Fig. 1). The Euryarchaeota genomes analyzed included: *Pyrococcus abyssi *from extremely thermophilic sulfur metabolizing archaea [52], *Methanococcus maripaludis *[53] from Methanococcales, *Halobacterium *sp. NRC-1 and *H. walsbyi *from extreme halophiles [54], *Thermoplasma acidophilum *and *Picrophilus torridus *belonging to the cell wall-less archaea [19,55], *Methanococcoides burtonii *from Methanosarcinales and *Methanopyrus kandleri *from the Methanopyrales order [56]. The chosen genomes should provide information regarding all archaeal proteins that are shared at a taxonomic level higher than a genus. The analysis of the remainder of the genomes, which was expected to provide information regarding proteins that are only unique to a given species, was not carried out.

Each ORF from these genomes was examined by means of blastp and PSI-blast searches against all available sequences from different organisms to identify proteins that are specific for only archaeal lineages. The methods and the criteria that we have used to identify proteins that are specific for either all or various subgroups of archaea are described in the Methods section. Generally, a protein was considered to be specific for a given archaeal lineage if all significant hits or alignments in the blastp and PSI-blast searches with the query protein were from the indicated group of archaeal species. In a few cases, where 1–2 isolated species from other groups also exhibited significant similarity, such proteins were retained as they provide interesting examples of lateral gene transfer (LGT) from archaea to other groups. Our analyses have identified 1448 proteins that are unique to different groups of Archaea and for which no homologues are generally found in any bacterial or eukaryotic species. Based on their specificity for different taxonomic groups, these proteins have been divided into a number of different groups (see Tables 2, 3, 4, 5, 6, 7 and Additional files). A brief description of the different subsets of archaeal-specific proteins and functional information regarding them, where known, is given below. In the description of these proteins that follows, the 'APE', 'HQ', 'Mbu', 'MK', 'MMP', 'PAB', 'PAE', 'PTO', 'Saci', 'Ta', 'VNG', and 'NEQ' part of the descriptors in proteins indicate that the original query protein sequence was from the genome of *A. pernix *K1, *H. walsbyi *DSM 16790, *M. burtonii *DSM 6242, *M. kandleri *AV19, *M. maripaludis *S2, *P. abyssi *GE5, *P. aerophilum *str. IM2, *P. torridus *DSM 9790, *S. acidocaldarius *DSM 639, *T. acidophilum *DSM 1728, *Halobacterium *sp. NRC-1 and *N. equitans*, respectively.

#### (a) Proteins that are specific for all Archaea

**Figure 2:**
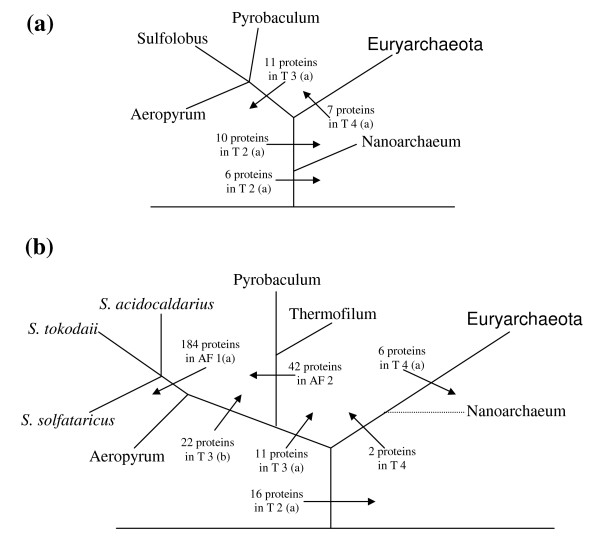
Interpretive diagrams showing the suggested evolutionary stages where genes for some of the signature proteins that are specific for the Crenarchaeota and Euryarchaeota as well as some of the Crenarchaeota subgroups, likely originated. The top diagram (A) indicates the evolutionary interpretation of the signature proteins in the absence of any other information, whereas that below (B) indicates our interpretation of this data taking into consideration other relevant information discussed in the text. The branching pattern shown here is unrooted and the proteins that are shared by all archaea were introduced in a common ancestor of all archaea. The dotted line for *N. equitans *in (B) indicates that its placement within Euryarchaeota lineage is uncertain. The abbreviations T and AF in these figures as well as others refer to tables and Additional files.

Table 2(a) shows a group of 16 proteins that are present in nearly all archaeal species but whose homologues are not found in any Bacteria or Eukaryotes with a single exception. Of these, the first 6 proteins in the left column (Table 2a) viz. PAB0063, PAB0252, PAB0316, PAB1633, PAB1716 and PAB2291, are present in all sequenced archaeal genomes. The observed E-values for these proteins from archaeal species are very low, close to 0, indicating that these proteins show very high degree of sequence conservation in various archaea. The unique presence of these proteins in all sequenced archaeal genomes indicates that these proteins could be regarded as distinctive characteristics or molecular signatures for the archaeal domain. The genes for these proteins likely evolved in a common ancestor of the Archaea and were then vertically acquired by other archaeal species. Makarova and Koonin [35] have also mentioned 6 proteins that are commonly shared by different archaea, but the identity of such proteins was not specified. These proteins are likely the same. The remaining 10 proteins in Table 2(a) are missing only in *N. equitans*, which is a tiny parasitic organism containing only 536 genes [57,58]. The species distribution pattern of these proteins can be accounted for by one of the following two possibilities. First, it is possible that *N. equitans *is the deepest branching lineage within archaea, as has been suggested [57,58] and the genes for these 10 proteins evolved in a common ancestor of the other archaea after its divergence (Fig. 2a). Alternatively, similar to the first 6 proteins, the genes for these 10 proteins evolved in a common ancestor of all archaea, but they were then selectively lost in *N. equitans *(Fig. 2b) [35,46,58]. Based upon our results, one cannot distinguish between these two possibilities. However, in view of the fact that the genome of *N. equitans *has undergone extensive genome shrinkage (only 0.49 Mb) and it is at least 3 times smaller than the next smallest archaeal genome (see Table 1), we favour the latter possibility (Fig. 2b) [35,46,58].

Of the proteins that are uniquely present in all archaea, PAB0063 corresponds to tRNA nucleotidyltransferase (CCA-adding enzyme), which builds and repairs the 3' end of tRNA [59]. Functionally similar enzymes are also present in bacteria and eukaryotes (assigned as Class II), but their sequences share very little homology with the archaeal CCA-adding enzyme (Class I), which explains why no homologs were detected in any bacteria or eukaryotes in blast searches. The main mechanistic difference between class I and class II enzymes is that the tRNA substrate is required to fully define the nucleotide binding site in class I enzyme, whereas class II has a preformed nucleotide binding site that recognizes CTP and ATP in the absence of tRNA [60]. Another protein PAB0316 is assigned as archaeal type DNA primase, which also has its synonymous counterparts in bacterial and eukaryotic species, but shows very little homology to them [61,62]. In the same way, protein PAB1633 is annotated as a PilT family ATPase, which showed very little similarity to bacterial ATPases involved in type IV pili biogenesis [54]. Further studies of this protein could provide insights into novel aspects of the archaeal flagellar system. A number of other proteins viz. PAB1716, PAB0018a, PAB0075, PAB0475 and PAB2104, have also been assigned putative functions based on sequence analysis, but their exact roles in archaeal cells remains to be determined. Interestingly, for protein PAB0075, two gene copies with acceptable E-values are also present in the genomes of *Dehalococcoides ethenogenes *195, *Dehalococcoides *sp. CBDB1 and *Dehalococcoides *sp. BAV1, which belong to Chloroflexi [2]. Because no homologue of PAB0075 is present in other bacteria, it is likely that this protein was transferred from archaea to the common ancestor of *Dehalococcoides *followed by a gene duplication event.

Table 2(b) lists 20 additional proteins, which are specific to archaea but missing in a small number of species. Because these proteins are present in most Euryarchaeota as well as Crenarchaeota species, but not detected in Bacteria or Eukaryotes except one LGT case (PAB2342, see note in Table 2), we consider them also to be distinctive characteristics of most Archaea. Of these proteins, 11 proteins (viz. PAB0654, PAB0950, PAB1135, PAB1906, PAB7388, PAB0547, PAB0552, PAB0623, PAB1272, PAB1429 and PAB1721) are mainly missing in the 4 Thermoplasmata species. Thermoplasmata are thermoacidophilic archaea which lack cell envelope [19,55,63](see Table 1). Some studies have suggested that high temperature and very low intracellular pH exert selective pressure favouring smaller genomes [19]. Thus, it is possible that genes for these proteins were selectively lost in the Thermoplasmata lineage. Most of these proteins are of unknown function. However, 8 of them have been assigned putative functions with the title of "archaeal type"'. For example, PAB0301 is archaeal sugar kinase, PAB0950 is archaeal transcription factor E α-subunit, PAB1387 is archaeal flagella accessory protein, PAB7094 is archaeal chromatin protein, and PAB0552 is archaeal type Holliday junction resolvase. These proteins do not show detectable sequence similarity to their counterparts in Bacteria or Eukaryotes, and some studies indicate that they also differ in terms of their structure, function or interaction with other cell components [64,65].

#### (b) Proteins that are specific for Crenarchaeota

As mentioned in the introduction, the Archaea are divided into 2 main groups, Crenarchaeota and Euryarchaeota, based on 16S rRNA trees as well many other gene trees and characteristics. The Crenarchaeota are also indicated to differ from Euryarchaeota in terms of their ribosome structure [30,31]. In comparison to Euryarchaeota, which contain physiologically and metabolically diverse groups of organisms, the Crenarchaeota were thought to be a pure collection of extreme thermophiles and most members metabolize sulfur. However, recent studies indicate that Crenarchaeota are much more diverse in their physiology and ecology than was previously believed [28,66]. Many species living in the cold ocean also belong to this group based on their branching pattern in 16S rRNA trees, although most of them have not been cultivated [67]. Currently, this phylum is comprised of one single class Thermoprotei containing three orders: Thermoproteales, Desulfurococcales and Sulfolobales. Fortunately, every order has a completely sequenced representative (see Table 1)[50,51,68,69], which provide a platform to explore the characteristics that are unique to crenarchaeal species. Comparative genomic surveys have revealed some molecular features that are shared by crenarchaea but not euryarchaea, such as the lack of histones, absence of the FtsZ-MinCDE system and distinctive rRNA operon organization [69]. Lake et al. have also identified distinctive differences in ribosome structure and an insert in elongation factor EF-G and EF-Tu, which can be used to distinguish Crenarchaeota from Euryarchaeota [6,30,70]. However, these features are not unique characteristics of the Crenarchaeota.

Blast searches on each ORF from the genomes of *A. pernix *and *S. acidocaldarius *DSM 639 [49,50] have identified 11 proteins which are shared by all five crenarchaeal species, but whose homologs are not found in other archaea, or any bacteria or eukaryotes with only 3 exceptions (see Table 3(a)). The genes for these proteins likely evolved in a common ancestor of the Crenarchaeota and they provide potential molecular markers for species from this phylum. Additionally, 22 proteins that are listed in Table 3(b) are only found in *A. pernix *and three *Sulfolobus *genomes. These proteins suggest that *Aeropyrum *and *Sulfolobus *may have shared a common ancestor exclusive of *Pyrobaculum*. However, we have also come across 9 proteins that are shared by *Aeropyrum *and *Pyrobaculum *(Table 3(c)) and 14 proteins that are exclusively present in the 3 *Sulfolobus *species and *Pyrobaculum *(see Table 3(d)). Hence, based upon the species distributions of these proteins, the relationships among the *Aeropyrum*, Sulfolobales and *Pyrobaculum *are not entirely clear (Fig. 2a). In phylogenetic trees Thermoproteales (i.e. *Pyrobaculum*) branches consistently earlier than Desulfurococcales (i.e. *Aeropyrum*) and Sulfolobales (Fig. 1) [32,44]. This observation in conjunction with the fact that *Aeropyrum *and Sulfolobus share larger numbers of proteins in common with each other suggests that these two groups likely shared a common ancestor exclusive of *Pyrobaculum *(Fig. 2b). The proteins that are only found in *Aeropyrum *and *Pyrobaculum*, or in *Sulfolobus *and *Pyrobaculum*, most likely evolved in a common ancestor of the crenarchaea, but were subsequently lost in either the Sulfolobales or *A. pernix *lineages.

In addition to these proteins that are uniquely present in either all sequenced Crenarchaeota genomes or different groups of Crenarchaeota species, these analyses have also identified 264 proteins that are unique for the Sulfolobales species (see Additional file 1). Of these, 184 proteins are present in all 3 sequenced *Sulfolobus *genomes, whereas the remaining 80 are present in at least two of the three *Sulfolobus *genomes. In this work, since blast analyses were not carried out on all three *Sulfolobus *genomes, it is likely that the numbers of genes or proteins that are uniquely shared by only two *Sulfolobus *genomes is much higher than indicated here. Chen et al. [50] have previously analyzed the genome of *S. acidocaldarius *DSM 639 and indicated the presence of 107 genes that were specific for Crenarchaeota and 866 genes that were specific to *Sulfolobus *genus. However, in the present work, relatively few genes that are uniquely shared by various Crenarchaeota species were identified. This difference could be due to more stringent criteria that we have employed for identification of proteins that are specific to different groups. The genome of *Thermofilum pendens *Hrk 5, which belongs to Thermoproteales, has also been partially sequenced and information for large numbers of genes/proteins from this species is available in the NCBI database. By carrying out blast searches on each ORF from *P. aerophilum *genome [51], we have identified 42 proteins that are only found in the above 2 Thermoproteales species (see Additional file 2). The numbers of proteins shared by these two species will likely increase once complete genome of *T. pendens *becomes available. Many of these proteins are expected to provide markers for the Thermoproteales order.

#### (c) Proteins that are specific for Euryarchaeota

The Euryarchaeota, which comprise a majority of the cultured and sequenced archaea, is a morphologically, metabolically and physiologically diverse collection of species as evidenced by the presence in this group of various methanogens, extreme halophiles, cell wall-less archaea and sulfate reducing microbes [2,13]. No unique biochemical or molecular characteristic that is commonly shared by all of the different lineages is known. The present study has identified 20 proteins that are only found in Euryarchaeota species with 3 exceptions (see Table 4). In this Table, the first 7 proteins (Table 4(a)) are present in most euryarchaeota species. Of these proteins, PAB0082 and PAB2404 were found in all sequenced euryarchaeota species. PAB2404 was also present in *N. equitans*, supporting its placement within the Euryarchaeota [35,46]. The protein PAB0082 is annotated as archaeosine tRNA-ribosyltransferase (ArcTGT), which catalyzes the exchange of guanine with a free 7-cyano-7-deazaguanine (preQ_0_) base, as the first step in the biosynthesis of an archaea-specific modified base, archaeosine (7-formamidino-7-deazaguanosine) [71]. It should be mentioned that there is another protein PAB0740 in the same genome, which is also annotated and experimentally confirmed as ArcTGT [72]. The latter belongs to a family of proteins that are highly conserved in all archaea species (including Crenarchaeota) and some bacteria. It seems that PAB0082 might be involved in RNA modification since it possesses a PUA domain (named after pseudouridine synthase and archaeosine transglycosylase), but its function is likely different from PAB0740. The protein PAB2404, which is annotated as DNA polymerase II large subunit, is highly conserved within Euryarchaeota, but is not found anywhere else except in *Nanoarchaeum*. This enzyme is the major DNA replicase in Euryarchaeota and also a distinctive molecular marker for this group [73,74]. The genes for the above proteins likely evolved in a common ancestor of Euryarchaeota (Fig. 2) and they provide molecular markers for this diverse group of organisms.

Another 13 proteins listed in Table 4(b) are found in almost all euryarchaeota, but they are missing in Thermoplasmata. Their distribution suggests that either Thermoplasmata is a deep branching lineage within Euryarchaeota or that the genes for these proteins have been selectively lost from Thermoplasmata [55]. Of these proteins, PAB0188 is also present in *N. equitans *supporting its placement with Euryarchaeota. Five other proteins from the first two columns in Table 4 (viz. MMP0243, Ta0062, VNG1263c, MMP1287, and VNG2408c) are also not found in the 4 Thermococci species. These results can again be explained by either selective loss of these genes from these particular groups or deeper branching of these lineages within the Euryarchaeota species. On the basis of proteins listed in Table 4, although one can infer that Thermoplasmata and Thermococci are deeper branching lineages within Euryarchaeota in comparison to methanogens, their relative branching order cannot be resolved.

#### (d) Proteins that are specific for different main groups within Euryarchaeota

##### Proteins specific for methanogenic archaea and their various subgroups

Currently, the methanogens form the largest group within the Euryarchaeota. They are distinguished from all other prokaryotes by their ability to obtain all or most of their energy via the reduction of CO_2 _to methane or by the process of methanogenesis. In the Bergey's manual [75], the methanogenes are divided into 5 distinct orders (viz. Methanobacteriales, Methanococcales, Methanomicrobiales, Methanosarcinales and Methanopyrales). Some studies have suggested that these organisms possess a set of unique enzymes which are responsible for methanogenesis, such as coenzyme M, Factor 420 and methanopterin [76]. However, no systematic study has been carried out thus far to identify proteins that are uniquely present in different methanogens. Our blast searches of proteins from different methanogens have led to identification of 31 proteins, which are uniquely found in various methanogenic archaea. Twenty of these 31 proteins are present in all sequenced methanogens, while 11 proteins are missing only in *M. stadtmanae*, which is a human intestinal inhabitant (see notes in Table 5). This archaeon generate methane by reduction of methanol with H_2 _and lacks many proteins present in the genomes of other methanogens [77,78]. Thus, it is highly likely that the 11 proteins missing in *M. stadtmanae *were selectively lost from this species. Therefore, it is very likely that the genes for these 31 proteins that are commonly shared by virtually all methanogens (Table 5(a)) evolved in a common ancestor of all methanogens.

**Figure 3:**
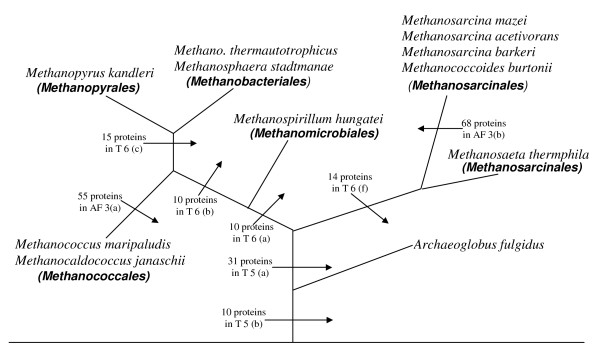
An interpretive diagram showing the evolutionary stages where genes for different proteins that are specific for methanogenic archaea likely originated. The 10 proteins that are uniquely shared by *A. fulgidus *and various methanogenic archaea indicate that this lineage is the closest ancestor of all methanogens.

These analyses have also identified 10 proteins that are uniquely shared by various methanogens as well as *A. fulgidus *(see Table 5(b)). The genes for these proteins likely evolved in a common ancestor of *A. fulgidus *and various methanogenic archaea and they point to a close relationship between these two groups of organisms (Fig. 3). Ten additional proteins are present in *A. fulgidus *as well as various Methanosarcinales and *M. hungatei *(Methanomicrobiales) (Table 5(c)). It is likely that the genes for these proteins also evolved in a common ancestor of *A. fulgidus *and various methanogenic archaea, but they were selectively lost in other methanogens. Of the proteins that are commonly shared by *A. fulgidus *and various methanogenic archaea, MMP0607 is reported to be a novel repressor of *nif *and *glnA *genes, which are involved in nitrogen assimilation [79]. Interestingly, 2 homologs of this protein are also found in 3 *Dehalococcoides *species, but nowhere else, which are very likely due to LGT. Protein MMP0984 is the ε-subunit of carbon-monoxide dehydrogenase complex, which is made up of five subunits in different methanogens [80]. The epsilon subunits are required for the reversible oxidation of CO to CO_2 _[81]. All of the other components could be found in a few bacterial species, while the ε-subunit is restricted to methanogenic archaea and *A. fulgidus *[82,83]. Protein MMP1499 is identified as a transcriptional regulator with a Helix-turn-helix (HTH) motif, but its exact role has not been reported.

Among the genes that are uniquely shared by various methanogenic archaea (or these archaea plus *A. fulgidus*), two large gene clusters responsible for methanogenesis are found. The proteins MMP1346, MMP1560–MMP1564 and MMP1566–MMP1567 (Table 5) are parts of an eight-component complex, coenzyme M methyltransferase (Mtr), which catalyzes an energy-conserving, sodium-ion-translocating step in methanogenesis from H_2 _and CO_2 _[84]. *M. maripaludis *contains all of the known Mtr subunits, but the gene coding for MtrF is fused into the N-terminal region of MtrA [53]. All other methanogenic archaeal genomes contain complete set of *mtr *genes. It is of interest to note that for the protein MMP1567 (MtrH), homologues with low E-values are also found in two *Desulfitobacterium hafniense *strains as well as in three Rhizobiales species (*Aminobacter lissarensis, Methylobacterium chloromethanicum*, and *Hyphomicrobium chloromethanicum*; α-proteobacteria) (see note in Table 5). These three rhizobiae species can use methyl halides as a sole source of carbon and energy, and all of them possess a set of *cmu *genes which are essential for methyl chloride degradation [85]. In particular, the CmuB protein which is homologous to MMP1567 transfers a methyl group to methylcobalamin:H_4 _folate (H_4_F), which is analogous to the reverse of the reaction catalyzed by MtrH in archaea [86]. In view of the sequence and functional similarity between MtrH and CmuB proteins, it is likely that the *mtrH *gene was laterally transferred from a methanogenic archaeon to the common ancestor of the above three rhizobiae species to serve the new functional role. The function of the laterally transferred *mtrH *related gene in *D. hafniense *is not known at present.

The proteins MMP1555–MMP1559 in Table 5 form another gene cluster, encoding the subunits of Methyl-coenzyme M reductase (MCR). This complex catalyzes the final reaction of the energy conserving pathway in which methylcoenzyme M and coenzyme B are converted to methane and the heterodisulfide CoM-S-S-CoB [87,88]. Except for these proteins, the other proteins listed in Table 5 are of putative or unknown functions. It is likely that these proteins are involved in some aspects of methanogenesis or other unknown pathways unique to methanogenic archaea. These proteins provide molecular markers for methanogens, which can be used for identification of new archaeal species capable of methane production.

The blast searches of the *M. maripaludis *[53] and *M. kandleri *[56] genomes have identified 10 proteins that are uniquely shared by all of the following species belonging to the orders Methanobacteriales (*M. thermoautotrophicus*), Methanococcales (*M. jannaschii*, *M. maripaludis*) and Methanopyrales (*M. kandleri*) (Table 6(b)). Of these, only 2 proteins are present in *M. stadtmanae*, which is also a Methanobacteriales that has lost most of its genes due to its adaptation to the human intestine [78]. The genes for these 10 proteins likely evolved in a common ancestor of the above groups of methanogens (Fig. 3), which corresponds to the cluster of methanogenic archaea referred to as "Class I methanogens" [13]. Interestingly, these studies have also identified 10 proteins that are uniquely shared by these methanogenic orders and *M. hungatei *(see Table 6(a)), which branches distantly in phylogenetic trees [13]. The unique presence of these proteins in these methanogens suggests that species from these groups shared a common ancestor exclusive of other methanogenic archaea (Fig. 3).

Fifteen additional proteins discovered in this work (Table 6(c)) are uniquely present in *M. kandleri *and various Methanobacteriales indicating that these two groups are more closely related to each other than the Methanococcales (Fig. 3). We have also come across 7 proteins that are uniquely shared by Methanococcales and Methanobacteriales (Table 6(d)), and 4 proteins that are only present in Methanococcales and Methanopyrales (Table 6(e)). The most likely explanation to account for the species distributions of these latter proteins is that their genes also originated in a common ancestor of the above three groups of methanogens, but were selectively lost in either the Methanobacteriales or Methanopyrales lineages. These analyses have also identified 14 additional proteins that are uniquely present in all 5 Methanosarcinales species (Table 6(f)), as well as 7 proteins that are only found in various Methanosarcinales and *M. hungatei *(Table 6(g)). Lastly, these studies have also identified 55 proteins that are uniquely present in *M. maripaludis *and *M. jannaschii *(Methanococcales, see Additional file 3(a)) and 68 proteins that are only present in *M. burtonii *and 3 *Methanosarcina *species, all belonging to the Methanosarcinaceae family (see Additional file 3(b)) (Fig. 3) indicating that they are likely distinctive characteristics of species from these groups.

Of the proteins that are uniquely found in Methanococcales, Methanobacteriales, Methanopyrales and Methanomicrobiales, 12 proteins viz. MMP1448–MMP1454, MMP1456, MMP1458–MMP1460 and MMP1467 are from a big gene cluster *eha*, which encodes the multisubunit membrane-bound [Ni-Fe] hydrogenase [89]. Two of these proteins, MMP1456 and MMP1458, are only found in *Methanococcales *(Table 6(e)). The whole *eha *operon is composed of 20 ORFs in the genome of *M. thermoautotrophicus *and of these only these 12 proteins are restricted to these methanogens while the other subunits have counterparts in bacteria. The precise roles of these 12 proteins, which are predicted to be integral membrane proteins in the hydrogenase complex, have not been determined [89]. Among the other proteins that are specific for these groups of methanogens, MMP0127 and MMP1716 are Hmd homologs, which catalyze the reversible dehydrogenation of N^5^, N^10^-methylenetetrahydromethanopterin [90]. In the proteins that are specific for the Methanococcales (see Additional file 3(a)), one large gene cluster (MMP0233–MMP0240) is found, but no information is available concerning its possible function. Except for these proteins, all other proteins that are specific for these methanogenic archaea are of unknown or putative function.

##### Proteins that are specific for Thermococci

Thermococci are obligately thermophilic, strictly anaerobic cocci, which are able to convert elemental sulfur to hydrogen sulfide. Thus, they are so called "extremely thermophilic sulfur metabolizer", which comprise one of the main functional groups within Euryarchaeota. According to the *Bergey's Manual *[75], the class Thermococci contains only one family, Thermococcaceae, consisting of 2 genera: *Thermococcus *and *Pyrococcus*. Currently, 4 species from this family have been completely sequenced (*Pyrococcus abyssi, P. horikoshii, P. furiosus and Thermococcus kodakarensis*; see Table 1) [52,91-93]. The blast searches on each protein from *P. abyssi *have identified 141 proteins that are shared by all 4 of these species (see Additional file 4(a)). All of these proteins show high degree of conservation within Thermococci and they do not have homologs in any other prokaryotes or eukaryotes except one possible LGT event (PAB1493, see note in Additional file 4). The genes for these proteins have likely evolved in a common ancestor of the Thermococci (Fig. 3). Of these proteins, PAB1510 is annotated as TBP-interacting protein (TIP), which forms complex with TBP (TATA-binding protein) to regulate transcription [94]. It is known that the archaeal transcription machinery is strikingly similar to that in eukaryotes [23], but no TBP-binding component was found in archaeal species until the discovery of the TIP in *T. kodakaraensis *[95,96]. Most other Themococci-specific proteins are of unknown function, although in a few cases limited similarity to domains in known protein families have been noted. A number of proteins (viz. PAB0643–PAB0644.1n; PAB1821–PAB1826) are clustered together in the *P. abyssi *genome, and it is possible that they may form functional units and are involved in related functions.

**Figure 4:**
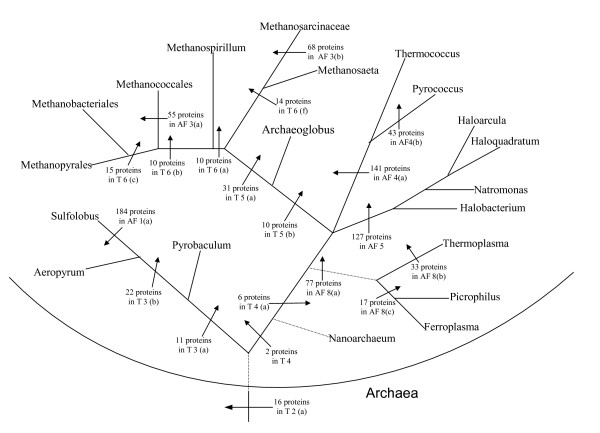
A summary diagram showing the branching order of different groups within archaea based upon species distribution patterns of various archaeal-specific proteins. The arrows mark the suggested evolutionary stages where proteins that are uniquely shared by the indicated groups were introduced. The details of these proteins can be found in the indicated tables (T) or Additional files (AF). The branching pattern shown here is unrooted. The dotted line for *N. equitans *indicates that its placement within Euryarchaeota is uncertain. The dotted line extending from the proteins found in all archaea indicates that one cannot use this to root the archaeal tree.

Cohen et al. [52] have reported a large number of proteins which are restricted to the *Pyrococcus *genus. However, a number of proteins from their list are also found in *T. kodakarensis *KOD1 [93], whose genome was not available when their work was published. Some proteins are not specific for either *Pyrococcus *or Thermococci according to our criteria and some of them are only found in one species – *P. abyssi*. Our analysis of the *P. abyssi *GE5 genome has also identified 43 proteins that are unique to the *Pyrococcus *genus (see Additional file 4(b)). Again, almost all of these proteins are of unknown function except PAB2241, which is annotated as RNase P, but this annotation seems arbitrary as it does not show significant sequence similarity to known RNases. The proteins that are uniquely found in the 3 *Pyrococcus *genomes likely evolved in a common ancestor of this genus (Fig. 4).

##### Proteins that are specific for Halobacteria

Extreme halophiles constitute another major class within Euryarchaeota. They require 5–10 times the salinity of seawater (ca. 3–5 M NaCl) for optimal growth [17,97]. In order to grow in such high salinity environments, they have developed a set of physiological adaptation, such as: high internal concentration of potassium chloride, acidic proteome with low pI value, high GC content with GC bias in the wobble position, unique chloride pumps to maintain osmotic balance, etc. [17,98,99]. Among archaea, halobacteria also have the unique ability to use solar energy to generate a proton gradient to synthesize ATP. So far, the Class Halobacteria harbors one family with 15 genera and 4 species have been completely sequenced, including *Halobacterium *sp. NRC-1, *Haloarcula marismortui*, *H. walsbyi *and *Natronomonas pharaonis *[54,98,100,101]. By performing blast searches on each protein in the *Halobacterium *sp. NRC-1 genome, we have identified 127 proteins, which are only present in all 4 Halobacteria species with only 3 exceptions (see Additional file 5).

Of the proteins listed in this Table, VNG0016H, VNG1096H, VNG2414H and VNG2563H are annotated as DNA-binding proteins or regulators because of the presence of HTH domain, but their exact functions have not been reported. VNG0667G is an ATP-binding protein of ABC transporter family. Several other proteins, such as VNG2089H and VNG2628H, have also been assigned possible functions based on weak similarity to known conserved domains in the CDD database [102], but their exact functions remain to be determined. Because of their high degree of conservation and uniqueness to halobacteria, the genes for these proteins likely evolved in a common ancestor of Halobacteria (Fig. 4) and they are presumably involved in unique physiological functions related to their adaptation to the hypersaline environment. Because of their specificity for Halobacteria, these proteins provide useful biomarkers for this group of species.

In addition to these proteins that are specific to all sequenced halobacterial species, we have also identified a large number of proteins either uniquely shared by 3 halobacterial species or only found in 2 halobacterial species (see Additional files 6 and 7). Surprisingly, these proteins are present in different combinations of halobacterial species. The four-halobacterial species are from 4 different genera within the Halobacteriales order and their relationships are unclear at present. The largest numbers of these proteins (i.e. 56) are uniquely shared by the *Haloarcula, Haloquadratum *and *Natronomonas *species, followed by 49 proteins that are restricted to *Haloquadratum *and *Haloarcula*. These results suggest that of these three species, *Haloquadratum *and *Haloarcula *are more closely related to each other and that *Halobacterium *might be the deepest branching of the four available halobacterial species (Fig. 4). However, the genome size of these halobacterial species varies and some of these protein sequences are present on plasmids found in these species, which makes it difficult to reliably infer their relationships solely based on the number of shared proteins. Among the proteins that are specific for halobacteria, only few have been assigned possible functions. Protein VNG2178H is annotated as PhiH1-like repressor and VNG0584H is assigned as a Rieske Fe-S protein. Two additional proteins VNG1720H and VNG2562H have been annotated as iron-binding proteins because of their similarity with FhuD and TroA_a domains, respectively [102]. All of the other proteins are of unknown function.

##### Proteins that are specific for Thermoplasmata

The *Thermoplasmata *group is comprised of cell wall-less archaea, which resemble the bacterial *Mycoplasma *species [63]. Generally, they are thermoacidophilic, aerobic or facultative anaerobic, and are able to reduce sulfur to H_2_S under anaerobic conditions [19,55]. To date, this class include three families-Thermoplasmaceae, Picrophilaceae, and Ferroplasmaceae, each represented by one genus [103,104]. Three complete genomes from this class (*T. acidophilum, T. volcanium *and *P. torridus*) are available at present (see Table 1) [19,55,63] and *Ferroplasma acidarmanus *Fer1 genome is draft assembled and sequence information for this is also available in the NCBI database. Our analyses have uncovered 77 proteins that are uniquely present in all four species belonging to this class (see Additional file 8(a)) (Fig. 4). Most of these proteins are present in all four available genomes, but a few are missing in one or two species, which is probably due to gene loss. Besides, we have also identified 33 proteins, which are shared only by the two *Thermoplasma *species (see Additional file 8(b)) and 17 proteins unique to *P. torridus *and *F. acidarmanus *(see Additional file 8(c)). The latter proteins indicate that species from Picrophilaceae and Ferroplasmaceae families are more closely related to each other (Fig. 4). All of these proteins are of unknown or predicted functions.

##### Proteins restricted to several archaeal lineages or showing sporadic distribution

In addition to the above proteins that are restricted to specific lineages of archaea, we have also identified 63 proteins, which are shared by several archaeal groups (see Table 7). The distribution pattern of these proteins could provide useful insights concerning the phylogenetic relationship between different groups. However, their distribution patterns could also be explained by gene losses in specific lineages or LGT between particular groups. Table 7 shows many proteins that are uniquely shared by various methanogenic archaea, Archaeoglobus and Thermococci. The first 5 proteins in Table 7(a) (PAB0076, PAB0138, PAB0965, PAB1927 and PAB1994) are present in all of the Thermococci and most of the methanogens. Four of these proteins are also present in *A. fulgidus*. The next 13 proteins in this Table are also uniquely found in most of the Thermococci as well as a number of methanogens and also in many cases in *A. fulgidus*. In addition, 6 proteins listed in Table 7(b) are only found in various Thermococci and *A. fulgidus*. These results suggest a closer relationship between the methanogenic archaea, *A. fulgidus *and Thermococci within the Euryarchaeota lineage. In conjunction with our earlier inference that *A. fulgidus *forms an outgroup of the methanogenic archaea, these results suggest that the above three groups are related in the following manner: Thermococci → *A. fulgidus *→ Methanogens.

Although the relationship suggested above is the most likely explanation for the observed results, we have also come across three proteins (VNG1263c, MMP11287 and VNG2408c) that are uniquely present in various Halobacteria, *A. fulgidus *and different methanogens. To account for their species distribution, one has to postulate that their genes have been selectively lost from the Thermococci. In addition, 9 proteins are only found in various Halobacteria as well as Methanosarcinales and Methanomicrobiales (Table 7(c)). Their distribution requires again either selective gene losses from other lineages or LGT from Halobacteria to these methanogens.

Our analyses have also uncovered 30 proteins that are uniquely shared by species from Thermoplasmata and *Sulfolobus *(see Table 7(d)). Among these proteins, 7 are present in all Thermoplasmata and *Sulfolobus *species for which sequence information is available, while the remainder are missing in 1 or more species. It has been reported that there has been much lateral gene transfer between *T. acidophilum *and *S. solfataricus*, both of which inhabit the same environment [55]. However, the shared presence of these proteins in these two groups could also result from a unique shared ancestry of these thermo-acidophilic archaea.

Another 43 Archaea-specific proteins are sporadically present in different archaeal species (see Additional file 9). A number of proteins in this group are present in a limited number (between 3 to 6) of archaeal species belonging to different groups. There are 2 possible explanations that can account for their sporadic distribution: First, it is possible that some of these genes are the remnants of sequences that also originated in an ancestral lineage of Archaea but they have been selectively lost in many species because they are not required for growth. Second, the sporadic presence of these genes in a number of archaeal species can also be explained if some of these genes originally evolved in a particular group or species of archaea and then transferred to other archaea by LGT [105]. However, in view of the observed specificity of these genes/proteins for archaea, the LGTs in these cases need to be selective and limited to within archaea.

## Conclusion

Comparative analyses of sequenced archaeal genomes presented here have led to identification of large numbers of proteins that are distinctive characteristics of either all archaea or its different main groups. Based upon these proteins, all of the main groups within Archaea (e.g. Crenarchaeota, Euryarchaeota, Halobacteria, Thermococci, Thermoplasmata, Methanogens) and their subgroups can now be clearly distinguished in molecular terms. The species distribution of these signature proteins strongly suggests that their genes have evolved or originated at various stages in the evolution of archaea, but once evolved, they are indicated to be generally stably retained in various descendents of these lineages with minimal gene loss or LGTs. Based upon the species distributions of these proteins, the evolutionary stages where the genes for these proteins have likely evolved are shown in Fig. 4. The evolutionary relationships among archaea have thus far been mainly inferred on the basis of their branching in phylogenetic trees based on 16S rRNA and certain protein sequences [2,7,13,23-25]. The results of our analyses although they support many inferences reached based on phylogenetic trees (viz. identification of all of the main clades in phylogenetic trees in molecular terms) (Fig. 1) [2,7,13,23-25], they also differ from them in important regards. In particular, our results shed important light on certain phylogenetic relationships that were very puzzling or were not resolved based on earlier studies. Some of these novel inferences are discussed below.

In phylogenetic trees based on 16S rRNA and various proteins sequences, the methanogenic archaea form at least two distinct clusters (see Fig. 1) [13,29,34,56,106]. In addition, in many of these trees, *M. kandleri *branches distinctly from all other methanogenic archaea [13,34,48]. The methanogenic archaea in these trees are interspersed by other groups of non-methanogenic archaea such as Halobacteriales, Archaeoglobus, Thermoplasmatales and Thermococcales (see Fig. 1) [13,34,48]. This has led to important questions concerning the origin of methanogenesis i.e. whether it evolved only once and its absence in the intervening lineages [13,29,35,76]. To account for these results, it has been suggested that methanogenesis evolved once in a common ancestor of the above groups, i.e. different methanogenic archaea, Halobacteriales, Archaeoglobus, Thermoplasmatales and also possibly Thermococcales, comprising virtually all euryarchaeota, but that the various genes involved in this process were subsequently lost from different groups except the methanogens [13,29,56]. This scenario, in essence, proposes that the common ancestor of different physiologically and metabolically distinct groups within euryarchaeota was a methanogen and this capability was independently lost in all other lineages.

In contrast to this proposal, our phylogenomics analyses have identified 31 proteins that are uniquely present in virtually all methanogens, as well as many proteins that are specifically shared by different subgroups of methanogens. Of these proteins only about 1/3 are indicated to be directly involved in methanogenesis and the cellular functions of others are presently not known. The unique presence of such large numbers of proteins by nearly all methanogens, but none of the above groups of archaea, strongly indicates that the genes for these proteins evolved in a common ancestor of various methanogens. These results strongly suggest that all methanogenic archaea form a mononphyletic lineage exclusive of all other groups of archaea (Fig. 4). Importantly, these studies have also identified 10 proteins that are uniquely shared by all methanogens as well as by *A. fulgidus*. In contrast, we have not come across any protein that various methanogenic archaea uniquely share with any of the Halobacterales or Thermoplasmatales. These observations are highly significant because they strongly suggest that Archaeoglobus and all of the methanogens shared a common ancestor exclusive of all other archaea. In other words, the ancestral lineage that led to the origin of methanogenesis very likely evolved from the Archaeoglobus lineage (Fig. 4). It is also significant that of the proteins that are uniquely shared by Archaeoglobus and methanogens, several form part of complexes that are important for nitrogen assimilation and methanogenesis. These results support the view that these characteristics have their origin within the Archaeoglobus lineage.

The present work also provides clarification regarding the phylogenetic position of *M. kandleri*. In phylogenetic trees based on 16S rRNA or different protein sequences, the branching of this species is highly variable [13,34,47,48] and it often forms the deepest branch within the Euryarchaeota. In the present work, we have identified 31 proteins that are uniquely shared by all methanogens including *M. kandleri*, as well as 10 proteins that *M. kandleri *specifically shares with various Methanobacteriales and Methanococcales, and 15 additional proteins that are only found in *M. kandleri *and the two Methanobacteriales species (*M. thermoautotrophicus *and *M. stadtmanae*). These observations reliably place *M. kandleri *with other methanogenic archaea with the Methanobacteriales as its closest relatives (Fig. 4). Our results also suggest a closer relationship of the Thermococcales to the Archaeoglobus and methanogenic archaea, although this relationship is not as strongly supported as between Archaeoglobus and Methanogens.

The observed differences in the evolutionary relationships among methanogens based upon phylogenomics analyses versus those by traditional phylogenetic methods can in principle be accounted for by three explanations. First, it is possible that the branching patterns of various clades in phylogenetic trees are misleading and they have been affected by factors such as long branch attraction effect [107,108]. Second, the polyphyletic branching of methanogens can also be explained (as indicated earlier) if the genes uniquely shared by all methanogens evolved in an early branching lineage such as *M. kandleri*, but subsequently they were either completely or partially lost from various non-methanogenic (viz. Halobacteriales, Thermoplasmatales and Archaeoglobus) groups that lie in between the two methanogenic clusters (Fig. 1). Third, lateral transfer of these genes from one methanogenic archaea to all others can also explain these results. Of these possibilities, we favour the first explanation, as the last two require extensive gene loss or LGT from (or into) multiple independent lineages.

The present work also supports the placement of *N. equitans *within the Euryarchaeota lineage. *N. equitans *has a very small genome (only 0.49 Mb), which is at least 3 times smaller than any other archaeal genome. Due to its very small size, there are only 6 genes that *N. equitans *uniquely shares with all other archaea. However, our analysis indicates that whereas *N. equitans *shares a few genes (PAB2404 and PAB 0188) with most of the Euryarchaeota, it does not share any gene uniquely with most of the Crenarchaeota species, indicating its closer affinity for the former lineage. Although our analysis of the *N. equitans *genome has not revealed any strong signals indicating its specific affinity for any of the Euryarchaeota groups, the shared presence of some proteins by *N. equitans *and Thermococci (and in some cases also *A. fulgidus *and methanogens) suggest that it may be related to the Thermococci. However, because of the extensive gene losses that have occurred in this genome, we are not able to draw any reliable inference in this regard. Therefore, although we have depicted *N. equitans *as a deep branching lineage within Euryarchaeota (Fig. 4), based upon our analysis, its placement within Euryarchaeota is not resolved.

The present work also suggests that Thermoplasmatales might be a deeper branching lineage within Euryarchaeota in comparison to the Thermococcales, Halobacteriales, Archaoglobous and Methanogens. This inference is suggested by the observation that a number of proteins that are uniquely present in almost all other Euryarcheota species are missing in the Thermoplasmatales. Although the absence of these proteins in the Thermoplasmatales can be explained by specific gene loss, the possibility that the genes for at least some of these proteins have evolved after the branching of Thermoplasmatales deserves serious consideration. The deeper branching of the Thermoplasmatales within the Euryarchaeota will place it closer to the Crenarchaeota. Such a placement could prove helpful in understanding why so many genes (i.e. 30) are uniquely shared by various Thermoplasmatales and the Sulfolobales.

For the archaeal-specific proteins identified in the present work, sequence information at present is available from only a limited number of archaeal species. Hence, it is important to obtain information for these genes/proteins from other archaeal species to confirm whether these proteins are distinctive characteristics of the specified groups or a subgroup of such species. These proteins in addition to their utility for phylogenetic and taxonomic studies also provide valuable means for understanding archaeal biology [35,38]. The cellular functions of most of these proteins are not known and further studies in this regard should prove very helpful in the discovery of novel biochemical and physiological characteristics that are unique to either all or different groups of archaea [38]. Lastly, the primary sequences of many of these genes/proteins are also highly conserved and they provide novel means for identification of different groups of archaea in various environmental settings by means of PCR amplification and other molecular biological and immunological methods.

## Methods

### Identification of Archaea-specific proteins

To identify proteins which are specific for Archaea or its various subgroups, all proteins in the genomes of *A. pernix *K1 (APE), *S. acidocaldarius *DSM 639 (Saci), *P. aerophilum *str. IM2 (PAE), *P. abyssi *GE5 (PAB), *M. maripaludis *S2 (MMP), *M. kandleri *AV19 (MK), *M. burtonii *DSM 6242 (Mbu), *Halobacterium *sp. NRC-1 (VNG), *H. walsbyi *DSM 16790 (HQ), *T. acidophilum *DSM 1728 (Ta) and *P. torridus *DSM 9790 (PTO), were analyzed. Protein-protein blast searches were carried out on each individual protein using the default parameters, without the low complexity filter, to identify different proteins where all significant hits were from archaea [109]. The results of blast searches were inspected for sudden increase in Expected values (E-values) from the last archaeal species in the search to the first non-archaeal organism. The proteins that were of interest generally involved a large increase in E-values from the last archaeal hit to the first hit from any other organism. Further, the E values of these latter hits were expected to be in a range higher than 10^-4^, which indicates a weak level of similarity that could occur by chance. However, higher E-values are sometimes acceptable for smaller proteins as the magnitude of the E-value depends upon the length of the query sequence.

All promising proteins identified by the above criteria were further analyzed using the position-specific iterated (PSI) blast program. In the present work, a protein was considered to be archaeal-specific if all hits producing significant alignments were from the indicated groups of archaeal species. However, we have also retained a few proteins where 1 or 2 isolated species from other groups (e.g. bacteria or eukaryotes) also had acceptable E-values. We consider these proteins to be also archaea-specific and their presence in isolated unrelated species is very likely due to lateral gene transfer. For all archaea-specific proteins described here, the protein ID, accession number and their possible functions (also COG or CDD number [102,110]) are presented in Tables 2, 3, 4, 5, 6, 7, 8 and Additional files 1, 2, 3, 4, 5, 6, 7, 8, 9. All proteins indicated in various tables are specific for the Archaea based on these criteria unless otherwise mentioned.

### Phylogenetic analyses

Phylogenetic analyses was carried out on a concatenated sequence alignment of 31 universally distributed proteins [45]. The information regarding these proteins is provided in the Additional file 10. For each of these proteins sequences from all 29 archaeal species were downloaded and multiple sequence alignments were created using ClustalX 1.83 program. A concatenated sequence alignment for all 31 proteins was imported into Gblocks 0.91b [111] to remove poorly aligned region. The resulting final alignment of 6,252 amino acid sites was used for phylogenetic analyses. A NJ tree based on this dataset was constructed by TREECON 1.3b program with Kimura two-parameter model distance [112]; Maximum-Likelihood tree were computed under a WAG+F model plus a gamma distribution with four categories by TREE-PUZZLE 5.2 [113,114]; Maximum-Parsimony tree were obtained by Mega 3.1 package [115]. All of the trees were bootstrapped 100 times.

## Authors' contributions

BG carried out blast searches on different proteins and was responsible for the initial evaluation of the results. RSG conceived and directed this study and was responsible for the final evaluation of the results. BG prepared a rough draft of the manuscript under RSG's directions, which was revised and modified by RSG. All authors have read and approved the final manuscript.

## Supplementary Material

Additional file 1 Proteins specific to Sulfolobales. These proteins are indicated to be specific for the either all three sequenced Sulfolobus species (*S. solfataricus, S. acidocaldarius *and *S. tokodaii*) or two of these based on Blastp and PSI-Blast searches. The proteins only found in a single *Sulfolobus *species are not listed here. Click here for file

Additional file 2 Proteins specific to *Pyrobaculum *and *Thermofilum*. For the proteins listed in this table, significant hits in Blastp and PSI-Blast searches are only observed for *Pyrobaculum *and *Thermofilum*. Because the genome of *Thermofilum pendens *is only partially sequenced, additional proteins of this kind may be found. Click here for file

Additional file 3 Proteins specific for particular groups of methanogens. For the proteins listed in this table, significant hits in Blastp and PSI-Blast searches are only observed for (a) *Methanococcales *and (b) *Methanosarcinaceae*. Click here for file

Additional file 4 Proteins specific for *Thermococci*. The proteins listed in this table are specific for either various (a) *Thermococci *(i.e. Thermococcus and Pyrococcus) or (b) various *Pyrococci*. Click here for file

Additional file 5 Proteins specific for various Halobacteria. The proteins listed in this Table are specifically found in various sequenced halobacteria species as determined by Blastp and PSI-Blast searches. Click here for file

Additional file 6 Proteins specific for various Halobacteria. These proteins are also specific for Halobacteria. However, unlike the proteins listed in Additional file 5, they are present in only three of the four sequenced halobacterial genomes. Click here for file

Additional file 7 Proteins specific for various Halobacteria. These proteins are also specific for Halobacteria but they are found in only two of the four sequenced halobacterial genomes. Click here for file

Additional file 8 Proteins specific for *Thermoplasmata*. These proteins are specific for either (a) all sequenced Thermoplasmata species, or (b) specific for only *Thermoplasma*, or (c) specific for *Picrophilus *and *Ferroplasma*. Click here for file

Additional file 9 Archaeal-specific proteins with sporadic distribution. These proteins are specific for Archaea but they show sporadic distribution in different groups. Click here for file

Additional file 10 List of proteins used in phylogenetic analysis Click here for file

## Figures and Tables

**Table 1:** Genome sizes, protein numbers and GC content of sequenced archaeal strains.

	Strain Name	Order	Temperature Range	Genome Size (Mb)	GC content (%)	Protein Number
*Crenarchaeota*	*Pyrobaculum aerophilum *str. IM2	*Thermoproteales*	H	2.22	52	2,605
	*Aeropyrum pernix *K1	*Desulfurococcales*	H	1.67	67	1,841
	*Sulfolobus acidocaldarius *DSM 639	*Sulfolobales*	A	2.23	36.7	2,223
	*Sulfolobus solfataricus *P2	*Sulfolobales*	A	2.99	35.8	2,977
	*Sulfolobus tokodaii *str. 7	*Sulfolobales*	A	2.69	32.8	2,825

*Euryarchaeota*	*Thermococcus kodakarensis *KOD1	*Thermococcales*	H	2.09	52	2,306
	*Pyrococcus abyssi *GE5	*Thermococcales*	H	1.77	42	1,898
	*Pyrococcus horikoshii *OT3	*Thermococcales*	H	1.74	42	1,955
	*Pyrococcus furiosus *DSM 3638	*Thermococcales*	H	1.91	42	2,125
	*Methanopyrus kandleri *AV19	*Methanopyrales*	H	1.69	60	1,687
	Methanothermobacter thermautotrophicus*	*Methanobacteriales*	T	1.75	49.5	1,873
	*Methanosphaera stadtmanae *DSM 3091	*Methanobacteriales*	M	1.77	27.6	1,534
	*Methanococcus maripaludis *S2	*Methanococcales*	M	1.66	33.1	1,722
	*Methanocaldococcus jannaschii *DSM 2661	*Methanococcales*	H	1.74	31.3	1,786
	*Methanospirillum hungatei *JF-1	*Methanomicrobiales*	M	3.54	45.2	3,139
	*Methanosaeta thermophila *PT	*Methanosarcinales*	T	1.9	53.5	1,696
	*Methanococcoides burtonii *DSM 6242	*Methanosarcinales*	M	2.58	45.8	2,273
	*Methanosarcina acetivorans *C2A	*Methanosarcinales*	M	5.75	42.7	4,540
	*Methanosarcina mazei *Go1	*Methanosarcinales*	M	4.10	41.5	3,370
	*Methanosarcina barkeri *str. fusaro	*Methanosarcinales*	M	4.87	39.2	3,624
	*Archaeoglobus fulgidus *DSM 4304	*Archaeoglobales*	H	2.18	46	2,420
	*Halobacterium *sp. NRC-1	*Halobacteriales*	M	2.57	65.9	2,622
	*Haloarcula marismortui *ATCC 43049	*Halobacteriales*	M	4.27	61.1	4,240
	*Haloquadratum walsbyi *DSM 16790	*Halobacteriales*	M	3.18	47.9	2,646
	*Natronomonas pharaonis *DSM 2160	*Halobacteriales*	M	2.75	63.1	2,822
	*Picrophilus torridus *DSM 9790	Thermoplasmatales	A	1.55	36	1,535
	*Thermoplasma acidophilum *DSM 1728	*Thermoplasmatales*	A	1.56	50	1,482
	*Thermoplasma volcanium *GSS1	*Thermoplasmatales*	A	1.58	50	1,499

	*Nanoarchaeum equitans *Kin4-M	N/A	H	0.49	31.6	536

Abbreviations for temperature range: H-hyperthermophilic; T-thermophilic; M-mesophilic; A-thermoacidophilic. * is strain *M. thermautotrophicus *str. Delta H.

**Table 2:** Proteins that are specific for all Archaea

*(a) Proteins specific to all Archaea*							
PAB0063	[NP_125796]	Cca	COG1746	PAB0247	[NP_126062]	DNA binding	COG1571
PAB0252	[NP_126069]	RNA-binding	CDD16214	PAB0439	[NP_126328]		COG1308
PAB0316	[NP_126166]	DNA primase	COG0358	PAB0475	[NP_126376]	regulator	COG1709
PAB1633	[NP_126790]	PilT ATPase	COG1855	PAB1040	[NP_127251]	SpoU	CDD6631
PAB1716	[NP_126666]	NMD3	CDD16276	PAB1106	[NP_127361]		CDD9578
PAB2291	[NP_125771]		CDD6629	PAB1706	[NP_126677]		COG1634
PAB0018a	[NP_125721]	RNA binding	COG2888	PAB2062	[NP_126118]		CDD16190
PAB0075^1^	[NP_125817]	dehydratase	CDD23288	PAB2104	[NP_126058]	HTH	COG1395

*(b) Archaea-specific proteins with gene loss in few species*							

PAB0301	[NP_126142]	SK	COG1685	PAB7388	[NP_127197]	Ribosomal_LX	CDD2437
PAB0654	[NP_126650]		CDD8168	PAB0469.1n	[NP_877631]		CDD8674
PAB0950	[NP_127106]	TFIIE	CDD480	PAB0547	[NP_126484]		COG1759
PAB1112	[NP_127373]		CDD5727	PAB0552	[NP_126501]	Hjr	CDD29957
PAB1135	[NP_127406]		CDD8168	PAB0623	[NP_126611]		CDD9586
PAB1241	[NP_127355]		CDD9682	PAB1272	[NP_127310]		COG1759
PAB1387	[NP_127161]	flaJ	COG1955	PAB1429	[NP_127105]		COG2433
PAB1715	[NP_126667]		CDD9801	PAB1721	[NP_126657]		COG2248
PAB1906	[NP_126377]		CDD2531	PAB2342^2^	[NP_125707]		CDD15774
PAB7094	[NP_126085]	Alba	CDD25844	PAB7309	[NP_126897]		CDD2523

These proteins were identified by BLASTP searches and their specificity is further confirmed by PSI-BLAST searches. For details, see method section. The protein ID number starting with PAB represents query protein from the genome of *P. abyssi *GE5, which was used as probe to perform the blast search. Accession numbers for these proteins are shown in square brackets. The possible cellular functions and COG or CDD number of some proteins are noted. For other proteins, the cellular functions are not known.

**Note ^1^**. Two low-scoring homologs to PAB0075 were also found in *Dehalococcoides ethenogenes *195 (*Chloroflexi*) and *Dehalococcoides *sp. CBDB1.

**Note ^2^**. A homolog to PAB2342 is also found in *Oenococcus oeni *PSU-1, *Leuconostoc mesenteroides *subsp. mesenteroides ATCC 8293 and *Clostridium perfringens *str. 13.

**Table 3:** Proteins that are specific for Crenarchaeota

*(a) Proteins specific to Crenarchaeota*					
APES019	[NP_147243]	ribonuclease p3	APE1241^2^	[NP_147816]	COG4343
APE0488	[NP_147273]	COG4914	APE1561	[NP_148025]	COG4900
APE0503	[NP_147284]	COG4755	APE1627^3^	[NP_148064]	CDD26669
APE0505^1^	[NP_147285]	CDD26165	APE1644	[BAA80645]	
APE0623	[NP_147373]	COG4888	APE1701	[NP_148108]	COG5494
APE0975	[NP_147640]	COG4879			

*(b) Proteins specific to Aeropyrum and Sulfolobus*					

APE0143	[NP_146996]	COG5491	APE1848	[NP_148210]	COG1259
APE0145	[NP_146997]		APE1936	[BAA80945]	
APE0168	[NP_147017]		APE1966	[NP_148294]	
APE0238	[NP_147072]		APE1996	[NP_148313]	
APE0429	[NP_147222]		APE2102	[NP_148384]	
APE0663	[NP_147399]	COG5431	APE2195	[NP_148451]	COG2083
APE0902	[NP_147588]		APE2325	[NP_148539]	
APE1113	[NP_147720]		APE2340	[NP_148552]	
APE1364	[NP_147897]		APE2435	[NP_148607]	COG4920
APE1626	[NP_148063]		APE2454	[BAA81469]	
APE1817	[NP_148186]	COG5399	APE2463	[NP_148628]	

*(c) Proteins specific to Aeropyrum and Pyrobaculum*					

APE0106	[NP_146969]		APE1230	[NP_147806]	
APE0730	[NP_147451]		APE1236	[NP_147812]	
APE0874	[NP_147564]		APE2409	[NP_148589]	
APE1194	[NP_147776]	COG5625	APE2602	[NP_148718]	
APE1228	[NP_147804]				

*(d) Proteins specific to Sulfolobus and Pyrobaculum*					

Saci_0004	[YP_254727]		Saci_1129	[YP_255774]	
Saci_0005	[YP_254728]		Saci_1813	[YP_256412]	COG4113
Saci_0035	[YP_254758]		Saci_1883	[YP_256481]	= Saci_1813
Saci_0223	[YP_254935]	CDD46009	Saci_2070	[YP_256657]	
Saci_0224	[YP_254936]	= Saci_0223	Saci_2080	[YP_256667]	= Saci_1813
Saci_0660	[YP_255337]		Saci_2195	[YP_256774]	= Saci_0223
Saci_0857	[YP_255517]		Saci_2357	[YP_256931]	= Saci_0223

The protein ID number starting with APE and Saci represents query protein from the genome of *A. pernix *K1 and *S. acidocaldarius *DSM 639. "=" means paralogous genes.

**Note ^1^**. A low scoring homolog to APE0505 is also found in *Ferroplasma acidarmanus *Fer1. **Note ^2^**. A low scoring homolog to APE1241 is also found in *Archaeoglobus fulgidus *DSM 4304.

**Note ^3^**. A low scoring homolog to APE1627 is also found in *Aquifex aeolicus *VF5.

**Table 4:** Proteins that are specific for Euryarchaeota

*(a) Proteins specific to almost all Euryarchaeota*					
PAB0082	[NP_125825]	Tgt COG1549	PAB2435	[NP_126297]	CDD25834
MMP0243*	[NP_987363]	CDD9595	P AB0315	[NP_126165]	CDD29150
PAB1089	[NP_127334]	COG2150	Ta0062*	[NP_393541]	CDD26662
PAB2404^1^	[NP_125813]	Pol II COG1933			

*(b) Proteins specific to Euryarchaeota except Thermoplasmata*					

PAB0161	[NP_125931]	COG1326	PAB1338	[NP_127222]	CDD9842
PAB0172	[NP_125944]	ATPase COG2117	PAB1517	[NP_126975]	COG1356
PAB0188^1^	[NP_125970]	CDD8172	PAB1804	[NP_126517]	CDD15772
PAB0951	[NP_127107]	COG4044	PAB2224	[NP_125887]	CDD5728
PAB1055^2^	[NP_127280]	COG4743	VNG1263c*	[AAG19620]	CDD2419
PAB1284	[NP_127297]	RecJ COG1107	VNG2408c*	[AAG20496]	COG3365
MMP1287*	[NP_988407]	CDD2419			

The protein ID number starting with MMP, Ta and VNG represents query protein from the genome of *M. maripaludis *S2, *T. acidophilum *and *Halobacterium *sp. NRC-1. * means protein is missing in the genomes of 4 *Thermococci *species.

**Note ^1^**. Homologs to PAB2404 and PAB0188 are also found in *Nanoarchaeum equitans *Kin4-M.

**Note ^2^**. Homolog to PAB1055 is also found in *Dehalococcoides sp*. CBDB1 and *D. ethenogenes *195.

**Table 5:** Proteins that are specific for methanogens (Methanoarchaeota)

*(a) Proteins specific to Methanoarchaeota*						
MMP0001	[NP_987121]	COG4014	MMP1346	[NP_988466]	MtrX	COG4002
MMP0021^5^	[NP_987141]	COG4079	MMP1555	[NP_988675]	MCR_B	CDD25889
MMP0143	[NP_987263]	COG4069	MMP1556	[NP_988676]	MCR_D	CDD3015
MMP0154	[NP_987274]	COG4070	MMP1557	[NP_988677]	MCR_C	CDD15906
MMP0311^5^	[NP_987431]	COG4048	MMP1558	[NP_988678]	MCR_G	CDD29638
MMP0312	[NP_987432]	COG4050	MMP1559	[NP_988679]	MCR_A	CDD8362
MMP0337	[NP_987457]	COG4029	MMP1560	[NP_988680]	MtrE	CDD9765
MMP0421	[NP_987541]	COG4052	MMP1561	[NP_988681]	MtrD	CDD9766
MMP0563^5^	[NP_987683]	COG4090	MMP1562	[NP_988682]	MtrC	CDD17461
MMP0642	[NP_987762]	COG4020	MMP1563	[NP_988683]	MtrB	CDD23666
MMP0656	[NP_987776]	COG4051	MMP1564^4^	[NP_988684]	MtrA	COG4063
MMP0665	[NP_987785]	COG4066	MMP1566	[NP_988686]	MtrG	CDD9769
MMP0698^5^	[NP_987818]	COG4033	MMP1593	[NP_988713]	COG1571	
MMP0701^5^	[NP_987821]	COG4081	MMP1644	[NP_988764]	COG4022	
MMP1223	[NP_988343]	COG4065	MMP1704	[NP_988824]	COG4008	
MMP1309^5^	[NP_988429]	COG4073				

*(b) Proteins specific to all methanogen and A. fulgidus*						

MMP0372	[NP_987492]	MTD CDD2518	MMP0962	[NP_988082]	COG4855	
MMP0400^1^	[NP_987520]	COG1707	MMP0976^5^	[NP_988096]	COG1810	
MMP0499^5^	[NP_987619]	ArsR CDD28947	MMP0984^5^	[NP_988104]	CO_dh	CDD3060
MMP0607^2^	[NP_987727]	NrpR COG1693	MMP1499^5^	[NP_988619]	HTH	COG4800
MMP0961^5^	[NP_988081]	CDD15263	MMP1567^3^	[NP_988687]	MtrH	CDD25859

*(c) Proteins specific to some methanogen and A. fulgidus*						

Mbur_0042	[YP_564815]		Mbur_0546	[YP_565273]		
Mbur_0348	[YP_565093]		Mbur_0652	[YP_565373]		
Mbur_0350	[YP_565095]		Mbur_0992	[YP_565682]		
Mbur_0545	[YP_565272]		Mbur_1754	[YP_566394]	CDD48145	
Mbur_0387	[YP_565131]	CDD28974	Mbur_1911	[YP_566543]		

The protein ID number starting with Mbur represents query protein from the genome of *M. burtonii*. "=" means paralogous genes.

**Note ^1^**. A homolog to MMP0400 is found in *Solibacter usitatus *Ellin6076 and *Rubrobacter xylanophilus *DSM 9941;

**Note ^2^**. A homolog to MMP0607 is found in *Dehalococcoides sp*. CBDB1 and *D. ethenogenes *195;

**Note ^3^**. A homolog to MMP1567 is found in 2 *Desulfitobacterium hafniense *strains (*Firmicutes*), and the CmuB protein from 3 species belonging to *Rhizobiales *of α-proteobacteria also show great similarity with MtrH;

**Note ^4^**. A homolog to MMP1564 is also found in *Dechloromonas aromatica *RCB;

**Note ^5^**. These 10 proteins are absent in the genome of *Methanosphaera stadtmanae *DSM 3091.

**Table 6:** Proteins that are specific to certain subgroups of methanogens

*(a) Proteins specific to Methanococcales, Methanobacteriales, Methanopyrales and Methanomicrobiales*								
MMP0125	[NP_987245]	COG4018			MMP1451	[NP_988571] EhaD	COG4039	
MMP0935	[NP_988055]	CDD26896			MMP1452	[NP_988572] EhaE	COG4038	
MMP1243	[NP_988363]	CDD30112			MMP1453	[NP_988573] EhaF	COG4037	
MMP1449	[NP_988569] EhaB	COG4041			MMP1454	[NP_988574] EhaG	COG4036	
MMP1450	[NP_988570] EhaC	COG4040			MMP1498	[NP_988618]	CDD26800	

*(b) Proteins specific to Methanococcales, Methanobacteriales and Methanopyrales*								

MMP0127	[NP_987247] Hmd	CDD8560			MMP1459	[NP_988579] EhaL	COG4035	
MMP0267	[NP_987387]	COG4053			MMP1497	[NP_988617]	COG4019	
MMP0618	[NP_987738]	COG4075			MMP1598	[NP_988718]	CDD15766	
MMP1217	[NP_988337]	COG4024			MMP1664	[NP_988784]	COG4071	
MMP1448	[NP_988568] EhaA	COG4042			MMP1716	[NP_988836] HmdII	CDD8560	

*(c) Proteins specific to Methanobacteriales and Methanopyrales*								

MK0046	[NP_613333]		MK0502	[NP_613787]		MK0927	[NP_614210]	
MK0108	[NP_613395]		MK0749	[NP_614033]		MK1599	[NP_614882] = MK0927	
MK0147	[NP_613434]		MK0750	[NP_614034]		MK1282	[NP_614565] = MK0502	
MK0241	[NP_613528]		MK0751	[NP_614035]		MK1513	[NP_614796]	
MK0431	[NP_613716]		MK0854	[NP_614137]	COG0707	MK1541	[NP_614824]	

*(d) Proteins specific to Methanosarcinales*								

Mbur_0178	[YP_564939]		Mbur_1314	[YP_565982]		Mbur_1890	[YP_566523]	
Mbur_0218	[YP_564978]		Mbur_1506	[YP_566163]		Mbur_1953	[YP_566584]	
Mbur_0544	[YP_565271]		Mbur_1512	[YP_566169]	COG4742	Mbur_1956	[YP_566587]	
Mbur_0997	[YP_565686]		Mbur_1689	[YP_566333]		Mbur_2254	[YP_566865]	
Mbur_1283	[YP_565952]		Mbur_1863	[YP_566496]				

*(e) Proteins only found in Methanococcales and Methanobacteriales*								

MMP0124	[NP_987244]		MMP1073	[NP_988193]	COG1320	MMP1460	[NP_988580] EhaM	
MMP0223	[NP_987343]		MMP1110	[NP_988230]	CDD2427	MMP1633	[NP_988753]	
MMP0940	[NP_988060]							

*(f) Proteins only found in Methanococcales and Methanopyrales*								

MMP1065	[NP_988185]		MMP1467	[NP_988587] EhaT		MMP1568	[NP_988688]	COG4010
MMP1118	[NP_988238]	CDD28974						

*(g) Proteins only found in Methanosarcinales and Methanomicrobiales*								

Mbur_0145	[YP_564912]		Mbur_1977	[YP_566606]		Mbur_2094	[YP_566718]	
Mbur_1266	[YP_565937]		Mbur_2017	[YP_566644]		Mbur_2402	[YP_567003]	
Mbur_1788	[YP_566426]							

The protein ID number starting with MK represents query protein from the genome of *M. kandleri *AV19.

**Table 7:** Proteins restricted to several archaeal lineages

*(a) Proteins only found in Thermococci, Archaeoglobus and methanogens*					
PAB0076	[NP_125818]	CDD15620	PAB1291	[NP_127284]	CDD41906
PAB0138	[NP_125896]	CDD9576	PAB1584	[NP_126876]	COG4072
PAB0965	[NP_127127]	CDD15705	PAB1860	[NP_126440]	
PAB1927^1^	[NP_126347]	CDD29323	PAB0813	[NP_126902]	COG1630
PAB1994	[NP_126245]	CDD9568	PAB0853	[NP_126970]	
PAB0036	[NP_125764]		PAB1251	[NP_127332]	endonuclease COG3780
PAB0054	[NP_125787]	CDD41919	PAB1779	[NP_126559]	CDD43950
PAB0176	[NP_125948]	CDD43579	PAB1806^2^	[NP_126515]	CDD43599
PAB1127	[NP_127394]	CDD30177	PAB2413	[NP_126288]	COG1710

*(b) Proteins unique to Thermococci + Archaeoglobus*					

PAB0981	[NP_127155]		PAB1672	[NP_126731]	
PAB0982	[NP_127156]		PAB3017	[NP_125737]	
PAB0985	[NP_127159]		PAB7298	[NP_126858]	

*(c) Proteins mainly shared by Halobacteria and some methanogens*					

VNG0240C^3^	[AAG18840]	COG4031	VNG2315H	[AAG20425]	MC1 CDD45747
VNG1236C	[AAG19598]		VNG2508C	[AAG20570]	Cyo COG4083
VNG1611C	[AAG19875]	COG4749	VNG2524H	[AAG20585]	
VNG1670C	[AAG19921]	COG3612	VNG2669G	[AAG20696]	
VNG1891H	[AAG20086]				

*(d) Proteins mainly shared by Thermoplasmata and Sulfolobus*					

Ta0035	[NP_393514]	COG5592	Ta1440	[NP_394894]	
Ta0164	[NP_393642]		Ta1453	[NP_394906]	
Ta0165	[NP_393643]		Ta1507	[NP_394957]	CDD29645
Ta0267	[NP_393747]	CDD43623	Saci_0040	[YP_254763]	
Ta0308	[NP_393788]		Saci_0054	[YP_254777]	
Ta0347	[NP_393826]	TauA CDD31059	Saci_0055	[YP_254778]	
Ta0547	[NP_394021]		Saci_0322	[YP_255031]	
Ta0548m^4^	[NP_394022]		Saci_0323	[YP_255032]	
Ta0583	[NP_394007]		Saci_0979	[YP_255633]	SdhD
Ta0759	[NP_394223]		Saci_1065	[YP_255715]	
Ta0793a	[NP_394256]		Saci_1491	[YP_256105]	CDD40171
Ta0938	[NP_394396]		Saci_1560	[YP_256166]	
Ta0939	[NP_394397]	PQQC CDD45213	Saci_1747	[YP_256346]	SoxE CDD46414
Ta1156	[NP_394612]		Saci_1952	[YP_256548]	
Ta1345	[NP_394801]		Saci_2078	[YP_256665]	

**Note ^1^**. A homolog to PAB1927 is also found in *Rubrobacter xylanophilus *DSM 9941;

**Note ^2^**. A homolog to PAB1806 is also found in *Aquifex aeolicus *VF5;

**Note ^3^**. A homolog to VNG0240c is also found in *Methanopyrus kandleri*;

**Note ^4^**. Two low-scoring homologs for Ta0548 are also found in *Gloeobacter violaceus *PCC 7421.
