# Supplementary material for: Phylogenomic analysis of proteins that are distinctive of Archaea and its main subgroups and the origin of methanogenesis
Source: BMC Genomics. 2007 Mar 29;8:86. doi: 10.1186/1471-2164-8-86 (PMC1852104; doi:10.1186/1471-2164-8-86)
Supplement: Additional file 1 — Proteins specific to Sulfolobales. These proteins are indicated to be specific for the either all three sequenced Sulfolobus species (S. solfataricus, S. acidocaldarius and S. tokodaii) or two of these based on Blastp and PSI-Blast searches. The proteins only found in a single Sulfolobus species are not listed here. [file 1471-2164-8-86-S1.pdf]

Additional File 1: Proteins specific to Sulfolobales

(a) Proteins specific to all 3 *Sulfolobus* species			
Saci_0014 [YP_254737]	Saci_0555 [YP_255243]	Saci_1215 [YP_255854]	Saci_1748 [YP_256347]
Saci_0022 [YP_254745]	Saci_0661 [YP_255338] rpoG	Saci_1228 [YP_255867]	Saci_1752 [YP_256351] COG5592
Saci_0028 [YP_254751]	Saci_0702 [YP_255375]	Saci_1229 [YP_255868]	Saci_1754 [YP_256353]
Saci_0045 [YP_254768]	Saci_0716 [YP_255388] CDD43579	Saci_1243 [YP_255882]	Saci_1808 [YP_256407]
Saci_0049 [YP_254772]	Saci_0744 [YP_255413]	Saci_1251 [YP_255890]	Saci_1811 [YP_256410]
Saci_0090 [YP_254810]	Saci_0746 [YP_255415]	Saci_1254 [YP_255893]	Saci_1832 [YP_256431] COG0727
Saci_0098 [YP_254816]*	Saci_0749 [YP_255418]	Saci_1267 [YP_255904]	Saci_1840 [YP_256439]
Saci_0099 [YP_254817]*	Saci_0783 [YP_255449]	Saci_1278 [YP_255913]	Saci_1845 [YP_256444]
Saci_0109 [YP_254827]	Saci_0816 [YP_255476]	Saci_1302 [YP_255934]	Saci_1854 [YP_256453]
Saci_0110 [YP_254828]	Saci_0845 [YP_255505]	Saci_1325 [YP_255953]	Saci_1855 [YP_256454] = Saci_0140
Saci_0130 [YP_254846]	Saci_0846 [YP_255506] CDD42003	Saci_1351 [YP_255979]	Saci_1856 [YP_256455] = Saci_0140
Saci_0131 [YP_254847]	Saci_0860 [YP_255520]	Saci_1354 [YP_255982]	Saci_1859 [YP_256458] cbsB
Saci_0140 [YP_254856] CDD46408	Saci_0870 [YP_255529]	Saci_1378 [YP_256004]	Saci_1863 [YP_256462]
Saci_0144 [YP_254858]	Saci_0878 [YP_255536]	Saci_1397 [YP_256020]	Saci_1886 [YP_256484]
Saci_0146 [YP_254860]	Saci_0879 [YP_255537]	Saci_1398 [YP_256021]	Saci_1895 [YP_256491]
Saci_0150 [YP_254864]	Saci_0881 [YP_255539]	Saci_1402 [YP_256025]	Saci_1912 [YP_256508]
Saci_0154 [YP_254868]	Saci_0895 [YP_255551]	Saci_1432 [YP_256054]	Saci_1917 [YP_256513]
Saci_0165 [YP_254878]	Saci_0898 [YP_255554]	Saci_1492 [YP_256106]	Saci_1933 [YP_256529]
Saci_0178 [YP_254890]	Saci_0912 [YP_255568]	Saci_1493 [YP_256107]	Saci_1988 [YP_256583]
Saci_0210 [YP_254922]	Saci_0913 [YP_255569]	Saci_1496 [YP_256110]	Saci_1992 [YP_256587] CDD28974
Saci_0215 [YP_254927]	Saci_0935 [YP_255590]	Saci_1504 [YP_256118]	Saci_2021 [YP_256610]
Saci_0218 [YP_254930]	Saci_0936 [YP_255591]	Saci_1506 [YP_256120] CDD30949	Saci_2044 [YP_256633]
Saci_0220 [YP_254932]	Saci_0937 [YP_255592]	Saci_1551 [YP_256157]	Saci_2048 [YP_256637] CDD43706
Saci_0232 [YP_254944]	Saci_0951 [YP_255606]	Saci_1573 [YP_256179]	Saci_2052 [YP_256639]
Saci_0255 [YP_254965]	Saci_0967 [YP_255622]	Saci_1590 [YP_256196]	Saci_2065 [YP_256652]
Saci_0274 [YP_254983] COG1468	Saci_0969 [YP_255624]	Saci_1591 [YP_256197]	Saci_2072 [YP_256659] = Saci_1676
Saci_0285 [YP_254994]	Saci_0970 [YP_255625]	Saci_1602 [YP_256208]	Saci_2082 [YP_256669] = Saci_0140
Saci_0287 [YP_254996]	Saci_0974 [YP_255629]	Saci_1615 [YP_256220]	Saci_2091 [YP_256678]
Saci_0298 [YP_255007]	Saci_0984 [YP_255638]	Saci_1627 [YP_256232]	Saci_2092 [YP_256679]
Saci_0304 [YP_255013]	Saci_0987 [YP_255641]	Saci_1634 [YP_256238]	Saci_2099 [YP_256686]= Saci_0360
Saci_0319 [YP_255028]	Saci_0989 [YP_255643]	Saci_1649 [YP_256252] COG5440	Saci_2103 [YP_256690]
Saci_0327[1] [YP_255036]	Saci_0990 [YP_255644]	Saci_1655 [YP_256258]	Saci_2107 [YP_256694] COG1011
Saci_0339 [YP_255048]	Saci_0996 [YP_255649]	Saci_1659 [YP_256262]	Saci_2108 [YP_256695]
Saci_0341 [YP_255050]	Saci_1012 [YP_255662]	Saci_1660 [YP_256263]	Saci_2158 [YP_256742]
Saci_0347 [YP_255056]	Saci_1029 [YP_255679]	Saci_1668 [YP_256271]	Saci_2194 [YP_256773]
Saci_0348 [YP_255057] CDD40783	Saci_1060 [YP_255710] =Saci_0140	Saci_1671 [YP_256274]= Saci_1185	Saci_2237 [YP_256816]
Saci_0352 [YP_255061]	Saci_1063 [YP_255713]	Saci_1673 [YP_256276]	Saci_2238 [YP_256817]
Saci_0354 [YP_255063]	Saci_1067 [YP_255717]	Saci_1676 [YP_256279]	Saci_2256 [YP_256835]
Saci_0360 [YP_255069]	Saci_1094 [YP_255741]	Saci_1678 [YP_256281] = Saci_1676	Saci_2257 [YP_256836]
Saci_0365 [YP_255074]	Saci_1159 [YP_255799]	Saci_1682 [YP_256285]	Saci_2258 [YP_256837]
Saci_0376 [YP_255085]	Saci_1171 [YP_255811]	Saci_1683 [YP_256286]	Saci_2264 [YP_256843]= Saci_2256
Saci_0426 [YP_255132]	Saci_1175 [YP_255815] flagellar	Saci_1686 [YP_256287]	Saci_2279 [YP_256858]
Saci_0431 [YP_255136]	Saci_1177 [YP_255817]	Saci_1687 [YP_256288]	Saci_2309 [YP_256888]
Saci_0436 [YP_255141]	Saci_1185 [YP_255825]	Saci_1689 [YP_256290]	Saci_2316 [YP_256894]
Saci_0439 [YP_255144]	Saci_1205 [YP_255844]	Saci_1737 [YP_256336]= Saci_1668	Saci_2349 [YP_256924]COG1331
Saci_0510 [YP_255209]	Saci_1206 [YP_255845]	Saci_1746 [YP_256345]	Saci_2368 [YP_256940]
(b) Proteins present in only 2 *Sulfolobus* species			
Saci_0002 [YP_254725]	Saci_0943 [YP_255598]	Saci_1415 [YP_256038]	Saci_1945 [YP_256541]
Saci_0021 [YP_254744]	Saci_0988 [YP_255642]	Saci_1433 [YP_256055]	Saci_1946 [YP_256542]
Saci_0116 [YP_254833]	Saci_1039 [YP_255689]	Saci_1434 [YP_256056]	Saci_1964 [YP_256560]
Saci_0132 [YP_254848]	Saci_1046 [YP_255696]	Saci_1435 [YP_256057]	Saci_1994 [YP_256589] = Saci_1945
Saci_0164 [YP_254877] CDD28974	Saci_1047 [YP_255697]	Saci_1446 [YP_256063]	Saci_2004 [YP_256596]
Saci_0167 [YP_254880]	Saci_1053 [YP_255703]	Saci_1452 [YP_256067]	Saci_2005 [YP_256597]
Saci_0172 [YP_254884]	Saci_1076 [YP_255726]	Saci_1527 [YP_256138]	Saci_2006 [YP_256598]
Saci_0284 [YP_254993]	Saci_1093 [YP_255740]	Saci_1631 [YP_256235]	Saci_2007 [YP_256599]
Saci_0314 [YP_255023]	Saci_1179 [YP_255819]	Saci_1692 [YP_256293]	Saci_2027 [YP_256616]
Saci_0460 [YP_255163] COG2129	Saci_1186 [YP_255826] MarR	Saci_1716 [YP_256315]	Saci_2038 [YP_256627]
Saci_0478 [YP_255177]	Saci_1225 [YP_255864]	Saci_1761 [YP_256360]	Saci_2086 [YP_256673] soxC
Saci_0485 [YP_255184]	Saci_1226 [YP_255865]	Saci_1766 [YP_256365]	Saci_2093 [YP_256680]
Saci_0495 [YP_255194]	Saci_1235 [YP_255874]	Saci_1776 [YP_256375]	Saci_2115 [YP_256702]
Saci_0499 [YP_255198]	Saci_1271 [YP_255907]	Saci_1791 [YP_256390]	Saci_2171 [YP_256754]
Saci_0668 [YP_255345]	Saci_1272 [YP_255908]	Saci_1815 [YP_256414]	Saci_2180 [YP_256759]
Saci_0679 [YP_255354]	Saci_1305 [YP_255936]	Saci_1833 [YP_256432]	Saci_2197 [YP_256776]
Saci_0680 [YP_255355]	Saci_1327 [YP_255955]	Saci_1898 [YP_256494]	Saci_2199 [YP_256778]
Saci_0733 [YP_255404]	Saci_1340 [YP_255968]	Saci_1928 [YP_256524]	Saci_2311 [YP_256890]
Saci_0794 [YP_255457]	Saci_1366 [YP_255993]	Saci_1929 [YP_256525]	Saci_2324 [YP_256902]
Saci_0938 [YP_255593]	Saci_1376 [YP_256002]	Saci_1932 [YP_256528]	Saci_2367 [YP_256939]

Note [1]. A low-scoring homolog to Saci_0327 is also found in *Aquifex aeolicus* VF5.
