# Supplementary material for: Phylogenomic analysis of proteins that are distinctive of Archaea and its main subgroups and the origin of methanogenesis
Source: BMC Genomics. 2007 Mar 29;8:86. doi: 10.1186/1471-2164-8-86 (PMC1852104; doi:10.1186/1471-2164-8-86)
Supplement: Additional file 2 — Proteins specific to Pyrobaculum and Thermofilum. For the proteins listed in this table, significant hits in Blastp and PSI-Blast searches are only observed for Pyrobaculum and Thermofilum. Because the genome of Thermofilum pendens is only partially sequenced, additional proteins of this kind may be found. [file 1471-2164-8-86-S2.pdf]

Additional File 2: Proteins specific to *Pyrobaculum* and *Thermofilum*

Gene ID, Accession Number and possible function		
PAE0111 [NP_558385]	PAE0993 [NP_559002] = PAE0531	PAE2275 [NP_559897]
PAE0307 [NP_558520]	PAE0719 [NP_558794] sdhD	PAE2276[1] [NP_559898] COG5277
PAE0335 [NP_558540]	PAE0826 [NP_558876]	PAE2659 [NP_560165] FdhE CDD44130
PAE0356 [NP_558556]	PAE0834 [NP_558883]	PAE2660 [NP_560166]
PAE0515 [NP_558657] = PAE0356	PAE0838 [NP_558887]	PAE2760 [NP_560237]
PAE0404 [NP_558586] = PAE0356	PAE1018 [NP_559020]	PAE0166 [NP_558425] = PAE2760
PAE0402 [NP_558585] = PAE0356	PAE1044 [NP_559041]	PAE2786 [NP_560253]
PAE1271 [NP_559182] = PAE0356	PAE1141 [NP_559097]	PAE2846 [NP_560297] = PAE2786
PAE0824 [NP_558875] = PAE0356	PAE1404 [NP_559282]	PAE2849 [NP_560300]
PAE0423 [NP_558599] = PAE0356	PAE1463 [NP_559318]	PAE2856 [NP_560304]
PAE3076 [NP_560470] = PAE0356	PAE1549 [NP_559373]	PAE2875 [NP_560321]
PAE2855 [NP_560303] = PAE0356	PAE1752 [NP_559518]	PAE2951 [NP_560381]
PAE0916 [NP_558939] = PAE0356	PAE1892 [NP_559615]	PAE3148 [NP_560526]
PAE0531 [NP_558661]	PAE1943 [NP_559650]	PAE3658 [NP_560895]

The protein ID number starting with PAE represents query protein from the genome of *Pyrobaculum aerophilum* str. IM2. “=” means paralogous genes.

Note [1]. A low scoring homolog to PAE2276 is also found in *Trypanosoma cruzi* strain CL Brener;
