# Supplementary material for: Phylogenomic analysis of proteins that are distinctive of Archaea and its main subgroups and the origin of methanogenesis
Source: BMC Genomics. 2007 Mar 29;8:86. doi: 10.1186/1471-2164-8-86 (PMC1852104; doi:10.1186/1471-2164-8-86)
Supplement: Additional file 3 — Proteins specific for particular groups of methanogens. For the proteins listed in this table, significant hits in Blastp and PSI-Blast searches are only observed for (a) Methanococcales and (b) Methanosarcinaceae. [file 1471-2164-8-86-S3.pdf]

Additional file 3: Proteins specific for particular groups of Methanogens

(a) Proteins specific to *Methanococcales*

Gene ID, Accession Number and possible function		
MMP0015 [NP_987135]	MMP0395 [NP_987515]	MMP1262 [NP_988382]
MMP0031 [NP_987151]	MMP0422 [NP_987542]	MMP1263 [NP_988383] CDD9511
MMP0032 [NP_987152] CDD28974	MMP0454 [NP_987574]	MMP1312 [NP_988432]
MMP0048 [NP_987168]	MMP0465 [NP_987585] CDD24258	MMP1447 [NP_988567] Repressor CDD28977
MMP0113 [NP_987233] CDD16462	MMP0528 [NP_987648]	MMP1456 [NP_988576] EhaI
MMP0141 [NP_987261] CDD6439	MMP0533 [NP_987653]	MMP1458 [NP_988578] EhaK
MMP0222 [NP_987342]	MMP0591 [NP_987711]	MMP1476 [NP_988596]
MMP0233 [NP_987353] CDD11699	MMP0646 [NP_987766]	MMP1530 [NP_988650]
MMP0234 [NP_987354] CDD15713	MMP0691 [NP_987811]	MMP1533 [NP_988653]
MMP0235 [NP_987355]	MMP0744 [NP_987864]	MMP1536 [NP_988656] COG4085
MMP0236 [NP_987356] CDD9580	MMP0751 [NP_987871] CDD12794	MMP1586 [NP_988706]
MMP0237 [NP_987357]	MMP0841 [NP_987961]	MMP1604 [NP_988724]
MMP0238 [NP_987358]	MMP0960 [NP_988080]	MMP1610 [NP_988730]
MMP0239 [NP_987359] CDD14419	MMP0992 [NP_988112] CDD26046	MMP1685 [NP_988805]
MMP0240 [NP_987360]	MMP1025 [NP_988145]	MMP1703 [NP_988823]
MMP0242 [NP_987362] CDD29645	MMP1078 [NP_988198]	MMP1719 [NP_988839]
MMP0262 [NP_987382] COG3286	MMP1125 [NP_988245]	MMP1720 [NP_988840] molybdate-binding
MMP0335 [NP_987455]	MMP1196 [NP_988316]	
MMP0344 [NP_987464]	MMP1228 [NP_988348]	

(b) Proteins specific to Methanosarcinaceae

Gene ID, Accession Number and possible function		
Mbur_0064 [YP_564835]	Mbur_0812[2] [YP_565518] MtaB	Mbur_1508 [YP_566165] = Mbur_1104
Mbur_0071 [YP_564842]	Mbur_0814 [YP_565520] = Mbur_0812	Mbur_1618 [YP_566272] = Mbur_0624
Mbur_0086 [YP_564856]	Mbur_0830 [YP_565534]	Mbur_1621 [YP_566275]
Mbur_0140 [YP_564907]	Mbur_0836 [YP_565540]	Mbur_1663 [YP_566313]
Mbur_0167 [YP_564928]	Mbur_0837 [YP_565541]	Mbur_1709 [YP_566351]
Mbur_0229 [YP_564989]	Mbur_0856 [YP_565557]	Mbur_1710 [YP_566352] = Mbur_0167
Mbur_0230 [YP_564990]	Mbur_0868 [YP_565569]	Mbur_1821 [YP_566457] = Mbur_1046
Mbur_0305 [YP_565057]	Mbur_1046 [YP_565729]	Mbur_1831 [YP_566464]
Mbur_0354 [YP_565099]	Mbur_1055 [YP_565738]	Mbur_1873 [YP_566506]
Mbur_0426 [YP_565164]	Mbur_1080 [YP_565760] = Mbur_1055	Mbur_1879 [YP_566512]
Mbur_0443 [YP_565180]	Mbur_1092 [YP_565772]	Mbur_1912 [YP_566544]
Mbur_0533 [YP_565261]	Mbur_1104 [YP_565782]	Mbur_1919 [YP_566551]
Mbur_0540 [YP_565267]	Mbur_1112 [YP_565789] CDD30278	Mbur_1968 [YP_566598]
Mbur_0610 [YP_565331] COG1470	Mbur_1114 [YP_565791]	Mbur_2053 [YP_566679]
Mbur_0624 [YP_565345]	Mbur_1115 [YP_565792] = Mbur_0533	Mbur_2060 [YP_566686]
Mbur_0673 [YP_565392] = Mbur_0610	Mbur_1193 [YP_565866]	Mbur_2083[4] [YP_566707] CDD42693
Mbur_0696 [YP_565411]	Mbur_1200 [YP_565873]	Mbur_2105 [YP_566729]
Mbur_0763[1] [YP_565474]	Mbur_1275 [YP_565946] = Mbur_0624	Mbur_2134 [YP_566757] CDD44427
Mbur_0769 [YP_565478]	Mbur_1325 [YP_565993]	Mbur_2312 [YP_566918] = Mbur_1366
Mbur_0775 [YP_565481]	Mbur_1372 [YP_566038] = Mbur_1325	Mbur_2346 [YP_566950]
Mbur_0781 [YP_565487]	Mbur_1366[3] [YP_566033]	Mbur_2368 [YP_566970]
Mbur_0782 [YP_565488] = Mbur_0781	Mbur_1371 [YP_566037] = Mbur_0769	Mbur_2428 [YP_567029]
Mbur_0805 [YP_565511]	Mbur_1462 [YP_566119] = Mbur_0443	

Note [1]. A homolog to Mbur_0763 is found in *M. maripaludis*;
Note [2]. A homolog to Mbur_0812 is found in *M. stadtmanae* and *Moorella thermoacetica*;
Note [3]. A homolog to Mbur_1366 is found in *Pelobacter carbinolicu.*
Note [4]. A homolog to Mbur_2083 is found in *Desulfitobacterium hafniense* Y51 and *D. hafniense* DCB-2.
