# Supplementary material for: Phylogenomic analysis of proteins that are distinctive of Archaea and its main subgroups and the origin of methanogenesis
Source: BMC Genomics. 2007 Mar 29;8:86. doi: 10.1186/1471-2164-8-86 (PMC1852104; doi:10.1186/1471-2164-8-86)
Supplement: Additional file 4 — Proteins specific for Thermococci. The proteins listed in this table are specific for either various (a) Thermococci (i.e. Thermococcus and Pyrococcus) or (b) various Pyrococci. [file 1471-2164-8-86-S4.pdf]

Additional file 4: Proteins specific for Thermococci

(a) Proteins specific to *Thermococci*		
PAB0019 [NP_125723]	PAB0644.1n [NP_877652]	PAB1556.1n [NP_877685]
PAB0037.2n [NP_877603]	PAB0649 [NP_126643]	PAB1570 [NP_126904]
PAB0055 [NP_125788] COG1822	PAB0672 [NP_126683]	PAB1571 [NP_126903]
PAB0070 [NP_125807]	PAB0705 [NP_126739]	PAB1585.1n [NP_877675]
PAB0079 [NP_125821]	PAB0711 [NP_126749]	PAB1599.1n [NP_877663]
PAB0114 [NP_125864]	PAB0720 [NP_126760] COG2886	PAB1634 [NP_126788]
PAB0116 [NP_125866]	PAB0729.1n [NP_877658]	PAB1635 [NP_126783]
PAB0131 [NP_125892] hydrolase COG1988	PAB0736 [NP_126791]	PAB1652.1n [NP_877656]
PAB0140 [NP_125898]	PAB0752 [NP_126817]	PAB1685 [NP_126706]
PAB0149 [NP_125910]	PAB0806.1n [NP_877679]	PAB1688 [NP_126703]
PAB0150 [NP_125911]	PAB0821 [NP_126918]	PAB1698 [NP_126692]
PAB0156 [NP_125923]	PAB0846 [NP_126960]	PAB1705 [NP_126678]
PAB0159 [NP_125929]	PAB0875 [NP_127005]	PAB1741.1n [NP_877651]
PAB0204 [NP_125993]	PAB0887 [NP_127021]	PAB1753 [NP_126596]
PAB0205 [NP_125994] CDD26426	PAB0931.1n [NP_877693]	PAB1794 [NP_126535] COG4933
PAB0207.1n [NP_877609]	PAB0940 [NP_127093]	PAB1807 [NP_126514] Adaptin_N CDD41644
PAB0227 [NP_126043]	PAB1030 [NP_127236]	PAB1818 [NP_126499]
PAB0258 [NP_126078]	PAB1039 [NP_127250] CDD22804	PAB1819 [NP_126498] ISOPREN CDD29787
PAB0275 [NP_126100]	PAB1075 [NP_127312] Dam COG0338	PAB1821 [NP_126496]
PAB0282 [NP_126120]	PAB1076 [NP_127313]	PAB1822 [NP_126495] CDD43938
PAB0294.1n [NP_877615]	PAB1093 [NP_127341]	PAB1823 [NP_126494]
PAB0328.2n [NP_877621]	PAB1094 [NP_127343]	PAB1824 [NP_126493]
PAB0330 [NP_126182]	PAB1096 [NP_127345]	PAB1825 [NP_126492]
PAB0385.1n [NP_877623]	PAB1110 [NP_127370] CDD3885	PAB1826 [NP_126491]
PAB0411 [NP_126289]	PAB1170 [NP_127455] CDD25595	PAB1851 [NP_126458]
PAB0416 [NP_126296] CDD26426	PAB1198 [NP_127422]	PAB1853 [NP_126451]
PAB0422 [NP_126303]	PAB1217 [NP_127397]	PAB1862 [NP_126438]
PAB0430.1n [NP_877625]	PAB1225.1n [NP_877721]	PAB1865 [NP_126435]
PAB0430.2n [NP_877626]	PAB1233 [NP_127371]	PAB1889 [NP_126407]
PAB0433 [NP_126320]	PAB1286 [NP_127294]	PAB1904 [NP_126380]
PAB0458 [NP_126354]	PAB1290 [NP_127285]	PAB1940 [NP_126329]
PAB0461.1n [NP_877627]	PAB1292.1n [NP_877711]	PAB1942 [NP_126325]
PAB0473 [NP_126374]	PAB1314.1n [NP_877708]	PAB1961 [NP_126280]
PAB0482.1n [NP_877633]	PAB1363 [NP_127189]	PAB2057 [NP_126126]
PAB0504 [NP_126421]	PAB1367 [NP_127185]	PAB2067 [NP_126113]
PAB0521 [NP_126455]	PAB1374 [NP_127174]	PAB2083 [NP_126089]
PAB0531 [NP_126469]	PAB1421 [NP_127120]	PAB2164 [NP_125977]
PAB0531.1n [NP_877638]	PAB1422 [NP_127119]	PAB2288.1n [NP_877604]
PAB0535 [NP_126473]	PAB1429.1n [NP_877695]	PAB2293 [NP_125767]
PAB0550.1n [NP_877641]	PAB1442 [NP_127085]	PAB2350 [NP_125697]
PAB0553 [NP_126502]	PAB1456 [NP_127066]	PAB2350.1n [NP_877596]
PAB0564.1n [NP_877645]	PAB1466 [NP_127053] COG1711	PAB2372 [NP_127103]
PAB0593.1n [NP_877647]	PAB1489.1n [NP_877692]	PAB2376.1n [NP_877704]
PAB0618 [NP_126606]	PAB1493[1] [NP_127013]	PAB2380 [NP_127126] TRP-repeat-containing
PAB0635 [NP_126626]	PAB1510 [NP_126987] TIP	PAB2405.1n [NP_877607]
PAB0643 [NP_126636]	PAB1512 [NP_126985]	PAB7217 [NP_126547]
PAB0644 [NP_126637] CDD24671	PAB1533 [NP_126952]	PAB7424 [NP_127347]
(b) Proteins specific to *Pyrococcus*		
PAB0005 [NP_125700]	PAB0747 [NP_126810]	PAB1428 [NP_127108]
PAB0014 [NP_125710]	PAB0820 [NP_126914]	PAB1609 [NP_126829]
PAB0038 [NP_125768]	PAB0831 [NP_126932]	PAB1621 [NP_126808]
PAB0052 [NP_125785]	PAB0834 [NP_126939]	PAB1623 [NP_126805]
PAB0053 [NP_125786]	PAB0847 [NP_126961]	PAB1632 [NP_126793]
PAB0246 [NP_126060]	PAB0850 [NP_126964]	PAB1650 [NP_126765]
PAB0312 [NP_126161]	PAB0940.1n [NP_877694]	PAB1663 [NP_126745]
PAB0324 [NP_126176]	PAB0984 [NP_127158]	PAB1758 [NP_126590]
PAB0438 [NP_126327]	PAB0998 [NP_127180]	PAB1981.1n [NP_877624]
PAB0590 [NP_126562]	PAB1069 [NP_127302]	PAB2009 [NP_126216]
PAB0621 [NP_126609]	PAB1080.5n [NP_877715]	PAB2111 [NP_126048]
PAB0622 [NP_126610]	PAB1117 [NP_127379]	PAB2241 [NP_125868] RnaseP
PAB0622.1n [NP_877650]	PAB1169 [NP_127457]	PAB2407.1n [NP_877610]
PAB0660 [NP_126662]	PAB1278 [NP_127304]	
PAB0726 [NP_126778]	PAB1373 [NP_127176]	

Note [1]. A low scoring homolog to PAB1493 is also found in *Thermoanaerobacter tengcongensis* MB4.
