# Supplementary material for: Phylogenomic analysis of proteins that are distinctive of Archaea and its main subgroups and the origin of methanogenesis
Source: BMC Genomics. 2007 Mar 29;8:86. doi: 10.1186/1471-2164-8-86 (PMC1852104; doi:10.1186/1471-2164-8-86)
Supplement: Additional file 5 — Proteins specific for various Halobacteria. The proteins listed in this Table are specifically found in various sequenced halobacteria species as determined by Blastp and PSI-Blast searches. [file 1471-2164-8-86-S5.pdf]

Additional file 5: Proteins specific to Halobacteria

Gene ID, Accession Number and possible function		
VNG0016H [AAG18656] COG2524	VNG1291H [AAG19639] CDD3145	VNG2163H [AAG20299] CopG
VNG0125H [AAG18748]	VNG1300H [AAG19646]	VNG2185H [AAG20320]
VNG0200C [AAG18811]	VNG1329H [AAG19668]	VNG2204H [AAG20334]
VNG0233H [AAG18835]	VNG1360H [AAG19691]	VNG2207H [AAG20337]
VNG0266H [AAG18859]	VNG1381H [AAG19707]	VNG2248H [AAG20369]
VNG0272H [AAG18864]	VNG1384H [AAG19710]	VNG2260H [AAG20379]
VNG0319H[1] [AAG18897]	VNG1403H [AAG19722]	VNG2306H [AAG20417]
VNG0359C [AAG18924]	VNG1417H [AAG19734]	VNG2335H [AAG20441]
VNG0429H [AAG18977]	VNG1448H [AAG19755]	VNG2340H [AAG20445]
VNG0488H [AAG19024] CDD25528	VNG1465G[3] [AAG19770] Brp	VNG2376H [AAG20471]
VNG0498C[2] [AAG19030] COG3270	VNG1479H [AAG19782] CDD12795	VNG2403H [AAG20492]
VNG0508H [AAG19040]	VNG1500H [AAG19798]	VNG2414H [AAG20501] CDD28974
VNG0604H [AAG19114]	VNG1530H [AAG19818]	VNG2415H [AAG20502]
VNG0659H [AAG19155]	VNG1558H [AAG19839]	VNG2440H [AAG20521]
VNG0660H [AAG19156] cox	VNG1559H [AAG19840]	VNG2442H [AAG20523]
VNG0667G [AAG19161] Trp4	VNG1591H [AAG19863]	VNG2490H [AAG20558]
VNG0677H [AAG19171]	VNG1625H [AAG19886] CDD10202	VNG2515H [AAG20577]
VNG0717H [AAG19198]	VNG1640H [AAG19896]	VNG2525H [AAG20586]
VNG0737H [AAG19216]	VNG1656H [AAG19911]	VNG2563H [AAG20613] COG4742
VNG0742H [AAG19219] COG4250	VNG1672H [AAG19922]	VNG2576H [AAG20622]
VNG0759H [AAG19233]	VNG1777H [AAG20001]	VNG2577C [AAG20623]
VNG0765H [AAG19238]	VNG1783H [AAG20006]	VNG2628H [AAG20663] NifU COG0694
VNG0768H[1] [AAG19241] CDD22832	VNG1788C [AAG20010]	VNG2641H [AAG20674]
VNG0789C [AAG19255]	VNG1798H [AAG20018]	VNG2656H [AAG20687]
VNG0798H [AAG19261] CDD9820	VNG1807H [AAG20024]	VNG2643H [AAG20676]
VNG0847H [AAG19297] CDD9915	VNG1809H [AAG20025]	VNG2674H [AAG20698]
VNG0858C [AAG19304] CDD10574	VNG1827H [AAG20036] CDD9399	VNG6082H [AAG20769]= VNG6377H
VNG0906H [AAG19340]	VNG1874C [AAG20074]	VNG6116H [AAG20794]
VNG0914H [AAG19346]	VNG1895H [AAG20089]	VNG6127H [AAG20802]
VNG0959H [AAG19382]	VNG1916H [AAG20105]	VNG6168H [AAG20838]= VNG6370H
VNG1002H [AAG19419]	VNG1920H [AAG20110]	VNG6171H [AAG20840]
VNG1003H [AAG19420]	VNG1934H [AAG20119]	VNG6319H [AAG20950]= VNG6171H
VNG1025H [AAG19437] CDD11933	VNG1989H [AAG20162]	VNG6357H [AAG20979] = VNG6116H
VNG1026H [AAG19438]	VNG1998H [AAG20169]	VNG6370H [AAG20989]
VNG1042H [AAG19451]	VNG2014H[1] [AAG20181] CDD22832	VNG6377H [AAG20994]
VNG1046H [AAG19453]	VNG2042H [AAG20201]	VNG6397H [AAG21008]= VNG6116H
VNG1052H [AAG19456]	VNG2044H [AAG20203] COG4194	VNG6402H [AAG21011] CDD28974
VNG1085H [AAG19483] CDD12933	VNG2059H [AAG20212]	VNG6444H [AAG21039] = VNG6377H
VNG1096H [AAG19493] CDD28974	VNG2081H [AAG20229]	VNG7130 [AAC82914]
VNG1220H [AAG19587]	VNG2089H [AAG20236] Rbn CDD8660	VNG7131 [AAC82915]
VNG1246H [AAG19606]	VNG2136G [AAG20274]= VNG1465G Blh	VNG7132 [AAC82916]
VNG1257H [AAG19615]	VNG2148H [AAG20285]	
VNG1289H [AAG19638]	VNG2157C [AAG20294] CDD2464	

The protein ID number starting with VNG represents query protein from the genome of *Halobacterium* sp. NRC-1.

Note [1]. VNG0319, VNG0768H and VNG2014H are paralogous genes, and a low scoring homolog to them is found in *M. hungatei* JF-1.

Note [2]. A homolog to VNG0498C is also found in *Methanosaeta thermophila* PT.

Note [3]. Two homologs to VNG1465G are also found in *Salinibacter ruber* DSM 13855.
