# Supplementary material for: Phylogenomic analysis of proteins that are distinctive of Archaea and its main subgroups and the origin of methanogenesis
Source: BMC Genomics. 2007 Mar 29;8:86. doi: 10.1186/1471-2164-8-86 (PMC1852104; doi:10.1186/1471-2164-8-86)
Supplement: Additional file 6 — Proteins specific for various Halobacteria. These proteins are also specific for Halobacteria. However, unlike the proteins listed in Additional file 5, they are present in only three of the four sequenced halobacterial genomes. [file 1471-2164-8-86-S6.pdf]

Additional file 6: Proteins specific to three Halobacteria species

(a) Proteins specific to *Haloarcula, Haloquadratum* and *Natronomonas*		
HQ1030A [YP_656818]	HQ1450A [YP_657222]	HQ2343A [YP_658080]
HQ1039A [YP_656827]	HQ1602A [YP_657372]	HQ2547A [YP_658267] = HQ2813A
HQ1053A [YP_656841]	HQ1655A [YP_657424]	HQ2569A [YP_658289]
HQ1055A [YP_656843]	HQ1666A [YP_657434]	HQ2670A [YP_658387]
HQ1086A [YP_656873]	HQ1684A [YP_657452]	HQ2722A [YP_658435]
HQ1087A [YP_656874]	HQ1702A [YP_657470]	HQ2813A [YP_658523]
HQ1112A [YP_656898]	HQ1709A [YP_657477]	HQ2870A [YP_658579]
HQ1115A [YP_656900]	HQ1738A [YP_657504]	HQ2998A [YP_658697] = HQ1086A
HQ1117A [YP_656902]	HQ1789A [YP_657554] = HQ2813A	HQ3026A [YP_658725]
HQ1186A [YP_656970]	HQ1947A [YP_657708] = HQ1312A	HQ3262A [YP_658954]
HQ1187A [YP_656971]	HQ2131A [YP_657885] CDD43604	HQ3440A [YP_659123] = HQ1450A
HQ1188A [YP_656972]	HQ2214A [YP_657963] = HQ1117A	HQ3500A [YP_659174]
HQ1223A [YP_657004]	HQ2215A [YP_657964]	HQ3505A [YP_659178] = HQ1087A
HQ1239A [YP_657019]	HQ2234A [YP_657983] = HQ1312A	HQ3506A [YP_659179] = HQ1086A
HQ1252A [YP_657032]	HQ2235A [YP_657984] = HQ1312A	HQ3578A [YP_659246] = HQ2131A
HQ1290A [YP_657068]	HQ2253A [YP_658000]	HQ3597A [YP_659264] = HQ1087A
HQ1312A[1] [YP_657090]	HQ2256A [YP_658003]	HQ3598A [YP_659265] = HQ1086A
HQ1331A [YP_657107]	HQ2273A [YP_658018] = HQ2215A	HQ3660A [YP_659321]
HQ1402A [YP_657175]	HQ2312A [YP_658054]	
(b) Proteins specific to *Haloarcula, Haloquadratum* and *Halobacterium*		
VNG0033H [AAG18673]	VNG1036H [AAG19446]	VNG2392H [AAG20483]
VNG0312H [AAG18892]	VNG1110C [AAG19502]	VNG2509H [AAG20571]
VNG0447H [AAG18990] CDD10432	VNG1345H [AAG19680]	VNG2532H [AAG20591]
VNG0578H [AAG19092]	VNG1447H [AAG19754]	VNG2634H [AAG20668]
VNG0579H [AAG19093]	VNG1459H [AAG19765]	VNG2645H [AAG20679]
VNG0581H [AAG19094]	VNG1538H [AAG19824]	VNG6355H [AAG20978]= VNG0033H
VNG0584H [AAG19097] QcrA CDD31067	VNG1849H [AAG20055] CDD30175	VNG6367H [AAG20987]
VNG0585H [AAG19098]	VNG1942H [AAG20126]	VNG6401H [AAG21010]= VNG0033H
VNG0587H [AAG19100]	VNG1991H [AAG20163]	VNG6418H [AAG21022]
VNG0590H [AAG19101]	VNG2262H [AAG20380]	VNG6419H [AAG21023]
VNG0911H [AAG19344]	VNG2342H [AAG20447]	
(c) Proteins specific to *Haloarcula, Natronomonas* and *Halobacterium*		
VNG0187H [AAG18800]	VNG1182H [AAG19557] CDD12795	VNG1865H [AAG20066]
VNG0199H [AAG18810]	VNG1183H [AAG19559] CDD25116	VNG2039H [AAG20199]
VNG0256H [AAG18852]	VNG1261H [AAG19618]	VNG2133H [AAG20272]
VNG0861H [AAG19307] CDD30165	VNG1372C [AAG19700] COG3975	VNG2178H [AAG20313] PhrH
VNG0978H [AAG19397]	VNG1380H [AAG19706]	VNG2379H [AAG20474]
VNG1034H [AAG19444]	VNG1621H [AAG19882] CDD12795	VNG2431C [AAG20515]
VNG1050H [AAG19455]	VNG1638H [AAG19895]	VNG2498H [AAG20563] CDD9693
VNG1064H [AAG19467] CDD1427	VNG1664H [AAG19916]	
VNG1087C [AAG19485] CDD8970	VNG1820H [AAG20031]	
(d) Proteins specific to *Haloquadratum, Natronomonas* and *Halobacterium*		
VNG0039H [AAG18678]	VNG0729H [AAG19207]	VNG2461H [AAG20537]
VNG0132C [AAG18753]	VNG1453H [AAG19759]	VNG6334H [AAG20962]
VNG0204H [AAG18812]	VNG1674H [AAG19924]	VNG7112 [AAC82896]
VNG0282H [AAG18870]	VNG1723H [AAG19962]	VNG7136 [AAC82920]
VNG0520H [AAG19048]	VNG2182H [AAG20317]	

The protein ID number starting with HQ represents query protein from the genome of *Haloquadratum walsbyi* DSM 16790.

Note [1]. A homolog to HQ1312A is also found in *Salinibacter ruber* DSM 13855.
