# Supplementary material for: Phylogenomic analysis of proteins that are distinctive of Archaea and its main subgroups and the origin of methanogenesis
Source: BMC Genomics. 2007 Mar 29;8:86. doi: 10.1186/1471-2164-8-86 (PMC1852104; doi:10.1186/1471-2164-8-86)
Supplement: Additional file 7 — Proteins specific for various Halobacteria. These proteins are also specific for Halobacteria but they are found in only two of the four sequenced halobacterial genomes. [file 1471-2164-8-86-S7.pdf]

Additional file 7: Proteins specific to two Halobacteria species

(a) Proteins specific to *Haloquadratum* and *Haloarcula*		
HQ1010A [YP_656797]	HQ1922A [YP_657684]	HQ2712A [YP_658425]
HQ1013A [YP_656800]	HQ2059A [YP_657815]	HQ2727A [YP_658440]= HQ2106A
HQ1031A [YP_656819]	HQ2092A [YP_657848]	HQ2759A [YP_658471]
HQ1032A [YP_656820]	HQ2106A [YP_657862] COG3609	HQ2778A [YP_658489]
HQ1083A [YP_656870] capC	HQ2111A [YP_657867]	HQ3188A [YP_658883]
HQ1141A [YP_656926]	HQ2123A [YP_657877]	HQ3404A [YP_659089]
HQ1459A [YP_657231]	HQ2124A [YP_657878]	HQ3475A [YP_659154]
HQ1606A [YP_657376]	HQ2129A [YP_657883]	HQ3482A [YP_659161]
HQ1612A [YP_657382]	HQ2136A [YP_657890]	HQ3572A [YP_659240]
HQ1623A [YP_657393]	HQ2138A [YP_657892]	HQ3582A [YP_659250]= HQ2136A
HQ1723A [YP_657491]	HQ2152A [YP_657904]	HQ3594A [YP_659261]
HQ1751A [YP_657516]	HQ2161A [YP_657912]	HQ3603A [YP_659270]
HQ1757A [YP_657522]	HQ2162A [YP_657913]	HQ3604A [YP_659271]
HQ1766A [YP_657531]	HQ2289A [YP_658032]	HQ3657A [YP_659318]
HQ1825A [YP_657591]	HQ2331A [YP_658068]	HQ4010A [YP_659406]= HQ2106A
HQ1889A [YP_657654]	HQ2435A [YP_658165]	
HQ1920A [YP_657682]	HQ2688A [YP_658404]	
(b) Proteins specific to *Halobacterium* and *Haloarcula*		
VNG0024H [AAG18664]	VNG1214H [AAG19582]	VNG1943H [AAG20127]
VNG0031H [AAG18671]	VNG1413H [AAG19731]	VNG1948H [AAG20131]
VNG0077H [AAG18713]	VNG1513H [AAG19806]	VNG1974H [AAG20150]
VNG0435H [AAG18981]	VNG1533H [AAG19820]	VNG2028H [AAG20191]
VNG0441H [AAG18987]	VNG1590H [AAG19862] CDD25792	VNG2035H [AAG20196]
VNG0492H [AAG19027] CDD12546	VNG1679H [AAG19928]	VNG2187H [AAG20322]
VNG0725H [AAG19204] CDD28974	VNG1720H [AAG19960] FhuD CDD29749	VNG2191H [AAG20325]
VNG0837H [AAG19291]	VNG1734H [AAG19969]	VNG2230H [AAG20353] CDD28974
VNG0945H [AAG19373]	VNG1758H [AAG19984]	VNG2399H [AAG20490] COG4711
VNG1047H [AAG19452]	VNG1894C [AAG20088]	VNG2562H [AAG20612] TroA_f CDD29742
VNG1056C [AAG19460]	VNG1910H [AAG20101]	VNG2566H [AAG20615]
VNG1095H [AAG19492]	VNG1942H [AAG20126]	VNG2678H [AAG20701]
(c) Proteins specific *Haloquadratum* and *Natronomonas*		
HQ1109A [YP_656895]	HQ2530A [YP_658250]	HQ3252A [YP_658944]
HQ1293A [YP_657071]	HQ2739A [YP_658451]	HQ3339A [YP_659026]= HQ2484A
HQ1658A [YP_657426]	HQ2748A [YP_658460]= HQ1109A	HQ3372A [YP_659058]
HQ1902A [YP_657665]	HQ2770A [YP_658482]	HQ3409A [YP_659094]
HQ2056A [YP_657812]	HQ2845A [YP_658555]	HQ3437A [YP_659120]
HQ2105A [YP_657861]	HQ2846A [YP_658556]= HQ2845A	HQ3607A [YP_659274]= HQ1902A
HQ2148A [YP_657900]	HQ2916A [YP_658623]	HQ3687A [YP_659348]
HQ2484A [YP_658207]	HQ3232A [YP_658925]= HQ2770A	
(d) Proteins specific to *Halobacterium* and *Natronomonas*		
VNG0041C [AAG18680]	VNG1119H [AAG19509]	VNG1619H [AAG19881]
VNG0373H [AAG18934]	VNG1130H [AAG19515]	VNG1642H [AAG19898]
VNG0509H [AAG19041]	VNG1268H [AAG19623]	VNG2619H [AAG20656]
VNG0708H [AAG19192]	VNG1423H [AAG19738]	VNG2633H [AAG20667]
(e) Proteins specific to *Halobacterium* and *Haloquadratum*		
VNG0738H [AAG19215]	VNG6031G [AAG20727] GvpC	VNG6420H [AAG21024]
VNG2244H [AAG20366]	VNG6284H [AAG20923]	VNG7127 [AAC82911]
VNG6023G [AAG20720] GvpI1	VNG6412H [AAG21019]	
