# Supplementary material for: Phylogenomic analysis of proteins that are distinctive of Archaea and its main subgroups and the origin of methanogenesis
Source: BMC Genomics. 2007 Mar 29;8:86. doi: 10.1186/1471-2164-8-86 (PMC1852104; doi:10.1186/1471-2164-8-86)
Supplement: Additional file 8 — Proteins specific for Thermoplasmata. These proteins are specific for either (a) all sequenced Thermoplasmata species, or (b) specific for only Thermoplasma, or (c) specific for Picrophilus and Ferroplasma. [file 1471-2164-8-86-S8.pdf]

Additional file 8: Proteins specific to Thermoplasmata

(a) Proteins specific to *Thermoplasmata*		
Ta0024 [NP_393503] CDD29957	Ta0678 [NP_394149] ArsR CDD28974	Ta1101 [NP_394560]
Ta0069 [NP_393548] COG1591	Ta0682[1] [NP_394153]	Ta1135 [NP_394592]
Ta0110a [NP_393589]	Ta0726a [NP_394194]	Ta1152 [NP_394608]
Ta0115 [NP_393593] CDD28974	Ta0743 [NP_394207]	Ta1174 [NP_394630]
Ta0150 [NP_393628]	Ta0754a [NP_394219]	Ta1180 [NP_394636]
Ta0160 [NP_393638] COG1630	Ta0784 [NP_394246]	Ta1184 [NP_394640] COG1814
Ta0178 [NP_393656]	Ta0828 [NP_394289]	Ta1218a [NP_394675]
Ta0222a [NP_393701]	Ta0843 [NP_394303]	Ta1220 [NP_394676]
Ta0271 [NP_393751]	Ta0855 [NP_394314]	Ta1235 [NP_394691]
Ta0305 [NP_393785]	Ta0859 [NP_394318]	Ta1238 [NP_394694]
Ta0314 [NP_393794]	Ta0867 [NP_394326]	Ta1302 [NP_394757]
Ta0336 [NP_393815]	Ta0916 [NP_394374]	Ta1306 [NP_394761]
Ta0337 [NP_393816]	Ta0918 [NP_394376]	Ta1317 [NP_394772]
Ta0354 [NP_393833]	Ta0921 [NP_394379]	Ta1321 [NP_394777]
Ta0367 [NP_393846] CDD2461	Ta0922 [NP_394380]	Ta1359a [NP_394816]
Ta0375a [NP_393855]	Ta0925 [NP_394383]	Ta1364 [NP_394820]
Ta0400 [NP_393879]	Ta0928 [NP_394386]	Ta1387 [NP_394841]
Ta0448 [NP_393926]	Ta0947a [NP_394407]	Ta1412 [NP_394866] COG2427
Ta0497 [NP_393974]	Ta0956 [NP_394416]	Ta1422 [NP_394876]
Ta0510 [NP_393986]	Ta0957 [NP_394417]	Ta1424 [NP_394878]
Ta0581 [NP_394067]	Ta0958 [NP_394418]	Ta1429a [NP_394883] HTH COG2512
Ta0590a [NP_394065]	Ta0993 [NP_394453]	Ta1454 [NP_394907]
Ta0592 [NP_394067]	Ta1007 [NP_394467]	Ta1483m [NP_394934]
Ta0606 [NP_394079]	Ta1028 [NP_394488]	Ta1491 [NP_394942]
Ta0615 [NP_394087]	Ta1047a [NP_394508]	PTO0559 [YP_023337]
Ta0665 [NP_394136]	Ta1065 [NP_394525]	PTO0902 [YP_023680]
**(b) Proteins specific to *Thermoplasma***		
Ta0012 [NP_393490]	Ta0377 [NP_393857] COG3432	Ta0757 [NP_394221]
Ta0032 [NP_393511]	Ta0401 [NP_393880] CDD29151	Ta0783 [NP_394245] HsdM CDD10160
Ta0109 [NP_393587]	Ta0408 [NP_393886]	Ta0798 [NP_394260]
Ta0168 [NP_393646]	Ta0409 [NP_393887]	Ta0846 [NP_394306]
Ta0173 [NP_393651]	Ta0412 [NP_393890]	Ta0874 [NP_394333]
Ta0209 [NP_393687]	Ta0438 [NP_393916]	Ta0982 [NP_394442]
Ta0210 [NP_393688]	Ta0449 [NP_393927]	Ta1207 [NP_394663]
Ta0231a [NP_393710]	Ta0503a [NP_393980]	Ta1215 [NP_394671]
Ta0233a [NP_393713]	Ta0584 [NP_394058]	Ta1236 [NP_394692]
Ta0254 [NP_393734] CDD11912	Ta0624 [NP_394096]	Ta1355 [NP_394811]
Ta0362 [NP_393841]	Ta0663 [NP_394134]	Ta1482 [NP_394933]
**(c) Proteins specific to *Picrophilus* and *Ferroplasma***		
PTO0047 [YP_022825]	PTO0810 [YP_023588]	PTO1183 [YP_023961]
PTO0196 [YP_022974]	PTO0876 [YP_023654]	PTO1236 [YP_024014]
PTO0396 [YP_023174]	PTO0940 [YP_023718]	PTO1294 [YP_024072]
PTO0452 [YP_023230]	PTO0952 [YP_023730]	PTO1325 [YP_024103]
PTO0749 [YP_023527]	PTO1082 [YP_023860]	PTO1374 [YP_024152]
PTO0781 [YP_023559]	PTO1173 [YP_023951]	

The protein ID number starting with Ta and PT represents query protein from the genomes of *Thermoplasma acidophilum* DSM 1728 and *Picrophilus torridus* DSM 9790.

Note [1]. A low-scoring homolog for Ta0682 is also found in *Symbiobacterium thermophilum* IAM 14863.
