# Supplementary material for: Phylogenomic analysis of proteins that are distinctive of Archaea and its main subgroups and the origin of methanogenesis
Source: BMC Genomics. 2007 Mar 29;8:86. doi: 10.1186/1471-2164-8-86 (PMC1852104; doi:10.1186/1471-2164-8-86)
Supplement: Additional file 9 — Archaeal-specific proteins with sporadic distribution. These proteins are specific for Archaea but they show sporadic distribution in different groups. [file 1471-2164-8-86-S9.pdf]

Additional file 9: Archaeal-specific proteins with sporadic distribution

Gene ID, Accession Number and possible function		
PAB0004 [NP_125699] NTP_transf COG2413	PAB1280 [NP_127301] CDD44167	PAB7281 [NP_126798] CDD44835
PAB0137 [NP_125895] COG4697	PAB1311 [NP_127260] CDD44179	APES038 [NP_147562]
PAB0502 [NP_126419] CDD29787	PAB1376 [NP_127172] CDD47265	APE0023 [NP_146907] COG4996
PAB0520 [NP_126454] PINc CDD47944	PAB1477 [NP_127036]	APE0158 [NP_147010]
PAB0531.2n [NP_877639]	PAB1556 [NP_126922]	APE0716 [NP_147438] COG4342
PAB0634 [NP_126625] ATPase CDD41674	PAB1596 [NP_126850]	APE0824 [NP_147521]
PAB0758a [NP_570852] COG4049	PAB1631 [NP_126794] COG4345	APE1123 [NP_147725] CDD47315
PAB0788 [NP_126861] PINc CDD47944	PAB1686 [NP_126705] CDD41923	APE1241 [NP_147816] CDD45915
PAB0880 [NP_127010] CDD44157	PAB1695 [NP_126695] COG4047	APE1539 [NP_148009]
PAB0921 [NP_127068]	PAB1697 [NP_126693] COG3368	APE1745 [NP_148138]
PAB0980 [NP_127154]	PAB2020 [NP_126204] CDD44179	APE2001 [NP_148317] RecB COG4080
PAB0981 [NP_127155] CDD46399	PAB2035.1n [NP_877616]	APE2057 [NP_148351]
PAB0982 [NP_127156] CDD46399	PAB3017 [NP_125737]	Ta1093a [NP_394553]
PAB0985 [NP_127159]	PAB7298 [NP_126858] COG5559	
PAB1054.1n [NP_877710]	PAB2252 [NP_125842] COG2403	
