# Supplementary material for: Phylogenomic analysis of proteins that are distinctive of Archaea and its main subgroups and the origin of methanogenesis
Source: BMC Genomics. 2007 Mar 29;8:86. doi: 10.1186/1471-2164-8-86 (PMC1852104; doi:10.1186/1471-2164-8-86)
Supplement: Additional file 10 — List of proteins used in phylogenetic analysis [file 1471-2164-8-86-S10.pdf]

Additional file 10: List of proteins used in phylogenetic analysis

Othologous Group	Average Length (aa)	Annotation
COG0012	397	GTP-binding protein, probable translation factor
COG0016	474	phenylalanyl-tRNA synthetase
COG0018	549	Arginyl-tRNA synthetase
COG0048	144	30S ribosomal protein S12
COG0049	193	30S ribosomal protein S7
COG0052	197	30S ribosomal protein S2
COG0060	1049	Isoleucyl-tRNA synthetase
COG0080	158	50S ribosomal protein L11
COG0081	215	50S ribosomal protein L1
COG0085	610	DNA-directed RNA polymerase subunit beta
COG0087	321	50S ribosomal protein L3
COG0091	155	50S ribosomal protein L22
COG0092	229	30S ribosomal protein S3
COG0093	132	50S ribosomal protein L14
COG0094	178	50S ribosomal protein L5
COG0096	131	30S ribosomal protein S8
COG0097	196	50S ribosomal protein L6
COG0098	198	30S ribosomal protein S5
COG0099	146	30S ribosomal protein S13
COG0100	133	30S ribosomal protein S11
COG0102	156	50S ribosomal protein L13P
COG0103	135	30S ribosomal protein S9
COG0184	152	30S ribosomal protein S15
COG0186	111	30S ribosomal protein S17
COG0197	174	50S ribosomal protein L10/L16
COG0200	154	50S ribosomal protein L15
COG0201	476	Preprotein translocase subunit SecY
COG0202	907	DNA-directed RNA polymerase subunit alpha
COG0256	189	50S ribosomal protein L18
COG0522	172	30S ribosomal protein S4
COG0533	540	O-sialoglycoprotein endopeptidase
